# Monoamine signaling and neuroinflammation: mechanistic connections and implications for neuropsychiatric disorders

**DOI:** 10.3389/fimmu.2025.1543730

**Published:** 2025-04-28

**Authors:** Shalini Saggu, Andrew Pless, Emily Dew, Destany Ware, Kai Jiao, Qin Wang

**Affiliations:** ^1^ Department of Neuroscience and Regenerative Medicine, Medical College of Georgia at Augusta University, Augusta, GA, United States; ^2^ Center for Biotechnology and Genomic Medicine, Medical College of Georgia at Augusta University, Augusta, GA, United States

**Keywords:** monoamines, norepinephrine, dopamine, serotonin, intracellular signaling, neuroinflammation, Alzheimer’s disease, major depression

## Abstract

Monoamines, including norepinephrine, serotonin, and dopamine, orchestrate a broad spectrum of neurophysiological and homeostatic events. Recent research shows a pivotal role for monoaminergic signaling in modulating neuroinflammation by regulating proinflammatory cytokines and chemokines within the central nervous system. Importantly, this modulation is not unidirectional; released proinflammatory cytokines markedly “feedback” to influence the metabolism of monoamine neurotransmitters, impacting their synthesis, release, and reuptake. This bidirectional interplay significantly links monoaminergic pathways and neuroinflammatory responses. In this review, we summarize current knowledge of the dynamic interactions between monoamine signaling and neuroinflammation, as well as their critical implications for the pathophysiology of neuropsychiatric disorders, including Parkinson’s Disease, Major Depressive Disorder, and Alzheimer’s Disease. By integrating recent findings, we shed light on potential therapeutic targets within these interconnected pathways, providing insights into novel treatment strategies for these devastating disorders.

## Graphical Abstract

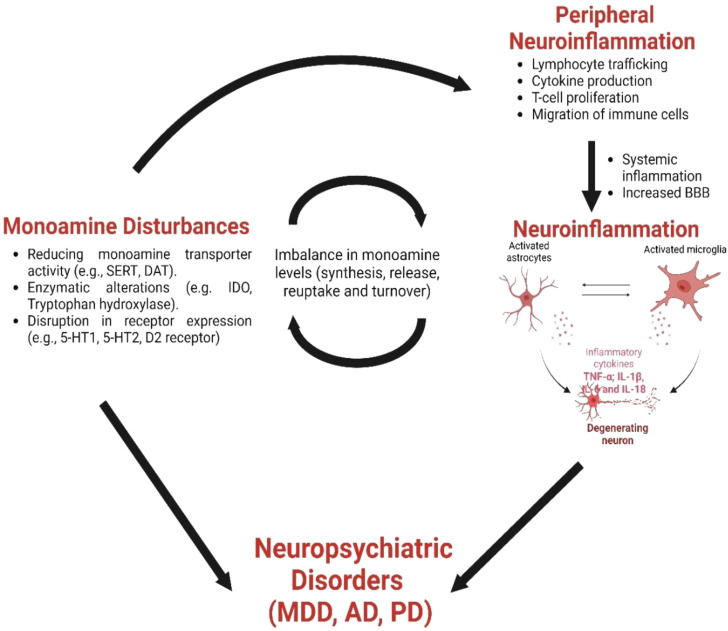

## 1 Introduction

Monoamine neurotransmitters, including norepinephrine (NE), serotonin (5-HT), and dopamine (DA), are important bioactive signaling molecules in the central nervous system (CNS). For example, monoaminergic (MA-ergic) systems regulate the gastrointestinal, respiratory, and cardiovascular systems, as well as modulate mood, cognition, sleep, nociception, temperature, perspiration, and other processes (1). Given their extensive influence, disruptions of these systems produce numerous pathological effects, contributing to the development of neuropsychiatric disorders (2–4).

MA-ergic systems participate in many physiological activities that regulate CNS inflammation (5), which can be initiated in response to various cues such as infection, traumatic brain injury, toxic metabolites, or autoimmune response (5). Neuroinflammation involves local microglial cells, infiltrating immune cells, and cytokines released from these cells and the peripheral nervous system (PNS). Upon infection or neuronal injury, microglia secrete either proinflammatory factors, which enhance cytotoxicity, or anti-inflammatory factors, which aid in wound healing and tissue repair (6). Excessive microglial activation, immune cell infiltration, and the release of proinflammatory cytokines can damage surrounding healthy neural tissue. Additionally, cellular factors released by dying neurons amplify chronic microglial activation and the infiltration of peripheral inflammatory mediators, accelerating further neuronal loss. Proinflammatory cytokines can also dysregulate MA-ergic systems by modulating their synthesis, release, and reuptake, further contributing to neuroinflammation, a common factor in many neurological disorders (7).

In this review, we explore recent findings on the interactions between MA-ergic function and neuroinflammation and briefly discuss the role of these interactions in neurological disorders such as Major Depressive Disorder (MDD), Alzheimer’s Disease (AD), and Parkinson’s Disease (PD). While histamine is also a monoamine involved in CNS function and neuroinflammation (8), this review focuses on NE, 5-HT, and DA due to their direct regulation of mood, cognition, and the pathophysiology of major neuropsychiatric disorders. These monoamine neurotransmitters are also the primary targets of many therapeutic interventions.

While many studies have linked neurological diseases to specific neuroinflammatory changes in the brain, we are continuously discovering that these diseases involve additional neuroinflammatory processes. Neuroinflammation plays a key role in the pathogenesis of depression, AD, and PD (9–11). By delineating the role of MA-ergic systems in regulating neuroinflammation and modulating monoamine neurotransmission by cytokines and chemokines, we endeavor to deepen our comprehension of how these systems contribute to disease pathophysiology. Our goal is to integrate existing knowledge to catalyze future research and the development of novel therapeutic approaches.

## 2 The monoamine system

The MA-ergic neurotransmitters NE, 5-HT, and DA play important roles in regulating functions within the nervous and immune systems by binding to receptors in the CNS and PNS. Neurochemical and structural changes in brain circuits, particularly in regions like the prefrontal cortex, amygdala, nucleus accumbens, and hippocampus, are linked to neuropsychiatric symptoms, such as depression, which is often associated with dysregulated 5-HT and DA signaling (4, 12, 13). Monoamine receptors, primarily G protein-coupled receptors (GPCRs), except for the 5-HT3 receptor (a ligand-gated ion channel) (14), are involved in cellular responses to neurotransmitters and the modulation of inflammation. GPCR subtypes exhibit distinct affinities for various ligands and influence physiological and inflammatory processes. Many immune cells express multiple GPCRs, regulating various inflammatory pathways (15, 16).

### 2.1 Norepinephrine

Adrenergic pathways are essential conduits for communication between the nervous and immune systems. NE is synthesized from DA by dopamine β-hydroxylase and is released from the locus coeruleus (LC), acting as a neuromodulator to regulate arousal, stress responses, anxiety, executive control, and memory consolidation. NE also plays a significant role in neuroinflammation, influencing microglia activation and the blood-brain barrier’s integrity (BBB) (17, 18). NE can be converted to epinephrine (EPI) (19), which is involved in the “fight-or-flight” response. Both EPI and NE stimulate adrenergic receptors, common receptors that are classified into three groups: α_1_ (α_1A_, α_1B_, α_1D_), α_2_ (α_2A_, α_2B_, α_2C_), and β receptors, all belonging to the G-protein-coupled receptor family.

α_1_ receptor subtypes activate phospholipase C, through Gα_q/11_ coupling, elevating levels of inositol triphosphate, Ca^2+^, and diacylglycerol. In contrast, α_2_ receptors inhibit adenylyl cyclase, via Gα_i/o_ coupling, decreasing cyclic adenosine monophosphate (cAMP) levels and protein kinase A (PKA) activity (20). Similarly, β receptors, which include three subtypes (β_1_, β_2_, and β_3_), also play an important role in adrenergic signaling. All β receptors associate with Gαs proteins and activate adenylyl cyclase, increasing cAMP levels and PKA signaling. Additionally, the β2 isoform can couple to Gαi/o, allowing for more diverse intracellular effects.

Adrenergic drugs, which target NE and EPI receptors, are commonly used in the treatment of psychiatric conditions. β-blockers like propranolol are sometimes used off-label to manage anxiety, although this is not an FDA-approved indication. Alpha-2 agonists like clonidine are prescribed for anxiety and ADHD, selective NE reuptake inhibitors (SNRIs) like venlafaxine increase NE levels to treat depression, and alpha-1 blockers like prazosin are used to address PTSD-related nightmares. Some of these uses, such as propranolol for anxiety, may not be officially approved but are utilized in clinical practice based on their effects.

### 2.2 Serotonin

5-HT, a vital neurotransmitter and hormone with diverse functions across various organs, is synthesized from tryptophan, with its rate-limiting step catalyzed by the enzyme tryptophan hydroxylase. 5-HT is primarily produced by enterochromaffin neuroendocrine cells in the gastrointestinal tract (21) and by serotonergic neurons in the raphe nuclei (located in the brainstem) within the CNS (22, 23). These serotonergic neurons are extensively distributed throughout the mammalian brain, making the serotonergic system the CNS’s largest, and perhaps the most complex, efferent system (24). Serotonergic nuclei, such as rostral, dorsal, and medial nuclei, diffuse their projections throughout the CNS, contributing to the regulation of temperature, appetite, sleep cycles, emesis, and sexual behavior. Conversely, caudal nuclei project into the spinal cord, modulating nociception and motor tone (4, 24, 25).

The functions of 5-HT in the CNS are diverse, affecting physiology, cognition, and behavior. Additionally, 5-HT has been implicated in CNS development, acting as a growth factor that guides the proliferation, organization, and maturation of the developing brain (26). It is also stored in blood platelets, highly concentrated in dense granules, and is released during agitation and vasoconstriction (27). Like many other neurotransmitters, 5-HT is usually taken back up by the presynaptic cell (reuptake) or degraded by monoamine oxidase (28). Selective 5-HT reuptake inhibitors (SSRIs), whose mechanism of action is to block 5-HT reuptake, are widely used to treat many psychiatric and mental health conditions (29).

Although only approximately 5% of total bodily 5-HT is found in the CNS, every brain cell is in close proximity to a serotonergic fiber, and all CNS regions express 5-HT receptors (5-HTRs) in a subtype-specific manner (26, 30). Individual neurons have been shown to express multiple 5-HTRs, leading to differential effects of 5-HT on the activity of distinct neurons (30). Furthermore, the serotonergic system interacts with other neurotransmitter systems, such as the catecholaminergic system, to influence numerous physiological processes (31).

5-HT’s actions are mediated by the 15 known receptor subtypes (across 7 mammalian 5-HT receptor classes), its interaction with the 5-HT transporter (SERT), and covalent binding to different effector proteins. Each family of 5-HT receptors has different subtypes, classified by morphology, pharmacologic profiles, and distribution (32). Despite the multitude of CNS functions influenced by 5-HT, its most clinically relevant aspect is its involvement in the pathophysiology of neuropsychiatric disorders.

### 2.3 Dopamine

DA is a neurotransmitter pivotal to brain function, governing movement control and reward-related behaviors while modulating cognitive functions through molecular substrates linked to plasticity. DA is synthesized from tyrosine through enzymatic reactions that convert it first into L-DOPA and then DA (33). Dysregulated DA signaling is implicated across various neurodegenerative, psychiatric, and autoimmune disorders, often accompanied by CNS neuroinflammation. Dopaminergic neurons, primarily found in specific brain regions such as the substantia nigra pars compacta, the ventral tegmental area (VTA), and the hypothalamus, delineate four principal pathways: the nigrostriatal, the mesolimbic, mesocortical, and the tuberoinfundibular pathways (34). The nigrostriatal pathway is fundamental for motor control, and its dysfunction is associated with PD, characterized by tremors, rigidity, and akinesia (35, 36). The mesocortical pathway, originating from the VTA, influences learning and memory (37) by projecting to various frontal cortex regions. The mesolimbic pathway, also originating from the VTA, regulates motivated behavior (36) through innervation of the ventral striatum, olfactory tubercle, and limbic system. On the other hand, the tuberoinfundibular pathway, originating from the hypothalamic periventricular and arcuate nuclei, modulates prolactin release and milk production.

DA mediates its effects through interactions with five DA receptors (DRs) on target cell membranes, categorized into D_1_-like (DRD1 and DRD5) and D_2_-like (DRD2, DRD3, and DRD4) receptor families (38). DRs, belonging to the GPCR class, activate downstream signaling pathways via heterotrimeric G proteins, specifically Gα_s/olf_, for stimulatory responses and Gα_i/o_, for inhibitory responses. Activation of D_1_ class receptors stimulates adenylate cyclase (AC), increasing cAMP levels, while D_2_-like receptors inhibit AC, decreasing cAMP levels (4, 39). Dopaminergic agonists, such as DA receptor agonists or drugs like pramipexole or ropinirole, mimic DA’s effect by directly activating these receptors (40). These drugs are often used in the treatment of conditions like PD and restless leg syndrome, where dopaminergic signaling is impaired. D1-like receptor agonists tend to increase cAMP levels, whereas D2-like receptor agonists may reduce cAMP levels, influencing neuronal activity and motor control (40, 41).

## 3 Monoamine signaling in peripheral inflammation

The immune and sympathetic nervous systems are deeply interconnected, with sympathetic nerve fibers innervating primary and secondary lymphoid tissues. These fibers primarily release NE, which regulates immune function by interacting with adrenergic receptors on immune cells, such as the β2-adrenergic receptor. This direct communication enables immune cells to respond to sympathetic signaling, influencing inflammation and immune responses (42). Beyond NE, other monoamines, such as 5-HT and DA, are key regulators of neuroinflammatory processes. Monoamine receptors are expressed on various immune cells such as macrophages, dendritic cells, and T cells, influencing immune function and inflammatory responses. Monoamine signaling influences cytokine secretion and other neuroimmune interactions, which are increasingly recognized as important factors in the development of neuropsychiatric and neurodegenerative diseases.

Further highlighting the overlap between the nervous and immune systems, neurons express pattern-recognition receptors such as Toll-like receptors, along with cytokine receptors. This enables neurons to respond directly to pathogen-associated molecular patterns (PAMPs) and immune-derived signals, further integrating immune and neuronal functions. Additionally, neurotransmitter signaling through monoamine receptors on immune cells helps regulate immune responses, reinforcing the bidirectional communication between these systems (43). This section explores how NE, 5-HT, and DA contribute to peripheral inflammation, with a specific focus on their roles in immune cell activation, cytokine secretion, and neuroinflammatory processes. Understanding these mechanisms provides valuable insight into immune regulation and the pathogenesis of neurological disorders.

### 3.1 Norepinephrine and peripheral inflammation

NE’s role in regulating peripheral immune responses has been extensively reviewed previously (43–46). NE can be converted into EPI by the enzyme phenylethanolamine N-methyltransferase, and is predominantly released from the adrenal glands, particularly from the adrenal medulla, into the bloodstream. NE and EPI can modulate lymphocyte trafficking, proliferation, and cytokine production, with both pro-inflammatory and anti-inflammatory effects depending on the immune cell type and receptor (45–49). For instance, NE and EPI activate β_2_ adrenoceptors, which inhibit proinflammatory cytokine production and promote anti-inflammatory cytokine production (45, 46). In contrast, NE and EPI acting on α_2_ adrenoceptors in monocytes and macrophages promote the production of tumor necrosis factor (TNF) and other cytokines (46, 50, 51). Interestingly, NE’s effects on β2 adrenoceptors can vary. In obese mice, β2 stimulation exerts anti-inflammatory effects, while in lean mice, it induces proinflammatory responses (49). Additionally, β2-adrenergic stimulation inhibits neutrophil functions, such as the formation of extracellular traps in human polymorphonuclear leukocytes (52).

### 3.2 Serotonin and peripheral inflammation

The role of 5-HT in inflammation and immunity has been widely studied and reviewed (5, 32). 5-HT, primarily synthesized in the gastrointestinal tract and released by platelets during inflammation (53, 54) also modulates immune responses. Notably, peripheral immune cells, including T cells, monocytes, macrophages, and dendritic cells, express one or more 5-HT receptors and possess 5-HT-related machinery transport (e.g. SERT), synthesis (via tryptophan hydroxylase 1), storage (in vesicles), and degradation (via monoamine oxidase). This allows 5HT to influence immune functions, including cellular activation, migration, and cytokine production.

5-HT signaling exerts complex modulatory effects on inflammation and immunity. The role of 5-HT in immune cells, particularly T cells, is of growing interest, as T cells play a central role in immune regulation and inflammation. 5-HT influences T cell responses by modulating T cell proliferation, differentiation, and cytokine production (55–58). It has been shown to promote the differentiation of naive T cells into Th1 or Th17 cells, both of which are involved in inflammatory responses, while its effects on regulatory T cells (Tregs) appear to be more context-dependent, sometimes promoting their differentiation and other times inhibiting it. While serotonergic components are well-documented in general T cell populations, the expression and function of specific 5-HTRs, such as 5-HT1, 5-HT2, 5-HT3, and 5-HT4, as well as SERT and enzymes, such as tryptophan hydroxylase, in T cell subtypes remain limited. The 5-HT1A receptor is known to be expressed on Tregs and has been implicated in promoting their anti-inflammatory functions. In contrast, 5-HT3 receptors, primarily expressed on effector T cells like Th1 and Th17 cells, have been linked to pro-inflammatory responses (5, 26, 59–61).

In addition to its role in regulating T cells, 5-HT regulates macrophage phagocytic function and cytokine secretion (62, 63), and it influences dendritic cell maturation and their ability to promote T-cell activation through modulating chemokine and cytokine release, particularly in response to microbial pathogens (64–67). Additionally, many 5-HT receptors expressed by immune cells have complex and sometimes opposing roles in inflammation. While 5-HT3 receptors are associated with the promotion of inflammation through increased production of pro-inflammatory cytokines, such as IFN-γ and IL-17, 5-HT2A and 5-HT4 receptors may attenuate these responses, possibly by dampening Th1 and Th17 differentiation or promoting Treg function (60, 61, 68). This receptor-specific dichotomy adds to the complexity of 5-HT’s role in modulating immune responses, making it difficult to generalize its pro- or anti-inflammatory effects.

Furthermore, discrepancies in study results and variations in experimental models, species, and even the timing of 5-HT exposure contribute to the ongoing uncertainty surrounding 5-HT’s immune functions. For example, previous research using chemical mitogens like Con A to activate T cells may not fully capture the complexity of *in vivo* activation, which typically involves interactions with antigen-presenting cells, co-stimulatory signals, and cytokine networks. Discrepancies in the effects of 5-HT on T cells could arise from its differential roles in effector T cells (e.g., Th1, Th17, CD8+ cytotoxic T cells) versus regulatory T cells, as these subsets exhibit distinct signaling pathways, such as IL-2 and mTOR, which are differentially regulated. Its influence on cAMP and mTOR signaling pathways is also likely to vary between effector and regulatory T cells, further complicating its effects. More targeted research is needed to clarify 5-HT’s role in different T cell subtypes, particularly in the context of Th17 and Treg cell interactions (5, 59, 61).

### 3.3 Dopamine and peripheral inflammation

DA has gained increasing attention for its role in modulating peripheral inflammation, as it both influences and is influenced by inflammatory processes. DA receptors on immune cells regulate the release of pro-inflammatory cytokines, shaping immune responses and contributing to the complex interplay between the nervous and immune systems. Many immune cells, including T cells, B cells, neutrophils, eosinophils, NK cells, dendritic cells, macrophages, microglia, and monocytes, are capable of producing DA at low levels and expressing DA receptors (69, 70). DA acts in an autocrine and paracrine manner, impacting immune cell functions through D1-like and D2-like receptors. These receptors regulate immune cell activation, inhibition, proliferation, and their specific immune functions. For example, DA can reduce reactive oxygen species (ROS) production and migration of human polymorphonuclear leukocytes by activating D1-like receptors, particularly D5R (71). DA also regulates various immune functions, such as cell differentiation, adhesion, migration, cytokine secretion, cytotoxicity, and chemotaxis. Its effects are complex, influencing immune homeostasis and disease, depending on immune cell type, receptor subtype, local concentration, and activation state. For instance, DA can activate normal resting peripheral human T lymphocytes, promoting cell adhesion, trafficking, and cytokine secretion (72–74), but it inhibits activated T cells in functions like cell proliferation, cytokine secretion, and cytotoxicity (75, 76).

DA has been shown to have anti-inflammatory effects, especially in the peripheral dopaminergic system, where activation of DA receptors modulates tissue inflammation and injury in conditions like acute pancreatitis and renal inflammation (77, 78). However, research on DA’s immune regulatory role has shown conflicting results due to variations in experimental models, samples, and methods. DA’s effects depend on factors like immune cell subtype, receptor abundance, and ligand availability, making comparisons across studies difficult (79). While extensive research has explored DA’s role in immune function (79, 80), its complex regulatory mechanisms and potential involvement in peripheral immune responses warrant further investigation.

## 4 Monoamine regulation of glial cell activation and neuroinflammation

Monoamines are important in regulating glial cell activation and neuroinflammation in the CNS. Glial cells, such as microglia and astrocytes, are essential for maintaining brain homeostasis and responding to stress, injury, or disease. Dysregulated monoamine signaling can lead to excessive activation of these glial cells, triggering neuroinflammation, which is linked to various neurological and psychiatric disorders (81–84). The effects of monoamines on glial function are complex, as they can influence microglial and astrocytic responses in both protective and detrimental ways. In addition, monoamine-induced peripheral inflammation may contribute to neuroinflammation as well. A deeper understanding of how NE, 5-HT, and DA regulate glial cells could open new therapeutic avenues for targeting neuroinflammation in these diseases. This section explores the roles of NE, 5-HT, and DA in glial activation and neuroinflammation, synthesizing key findings on their impact.

### 4.1 Norepinephrine

Adrenergic receptors are present in varying densities among different types of glial cells (85, 86). Through these receptors, NE mainly exerts anti-inflammatory effects on the CNS. Multiple evidence supporting an anti-inflammatory role of NE come from the neurotoxin N-(2-chloroethyl)-N-ethyl-2-bromobenzylamine (DSP4)-treated animals. DSP4 treatment induces degeneration of central noradrenergic pathways, augments the proinflammatory response to β-amyloid in rat cortex, significantly elevating iNOS, interleukin-beta (IL-1β), and interleukin-6 (IL-6) expression (87). The heightened IL-1β expression in microglia and astrocytes after β-amyloid injection in DSP4-treated rats was mitigated by co-injection with NE or the β-adrenergic receptor agonist isoproterenol. Depletion of NE in aged rats by DSP4, followed by LPS injection to induce systemic inflammation, also increased serum levels of several proinflammatory cytokines, augmented astroglial and microglial activation in the hippocampus, and decreased cognitive performance in a novel object recognition task (88). Similarly, injecting mice with DSP4 induced chronic neuroinflammation and neurodegeneration (89, 90). On the other hand, increasing NE by pharmacological SNRIs, such as desipramine and atomoxetine, enhanced central noradrenergic tone and reduced proinflammatory cytokines IL-1β and TNFα, as well as iNOS and microglial activation markers, in the rat cortex and hippocampus following LPS challenges (91, 92). Desipramine and atomoxetine have also been reported to induce IL-10 and suppressor of cytokine signaling-3 in the rat cortex and hippocampus (93).

β-adrenergic receptors primarily mediate the anti-inflammatory role of NE, and their activation has been shown to reduce proinflammatory responses in microglia and astrocytes induced by inflammatory stimuli such as LPS (92, 94–97). Administration of a β_1_ adrenergic receptor agonist, xamoterol, suppressed LPS-induced TNF-α production in rat microglia cultures, reducing proinflammatory markers, and attenuating microgliosis and astrogliosis. A β_2_ adrenergic receptor agonist, clenbuterol, was also able to mitigate LPS-induced expression of TNF-α, IL-6, chemokines RANTES and IP-10, and cell adhesion molecules in rat cortex (98).

NE activation of β-adrenoceptors can exert anti-inflammatory effects by inhibiting the expression of interferon-gamma (IFN-γ)-induced major histocompatibility complex class II molecules (99, 100). Additionally, NE promotes the expression IL-1 receptor antagonist (IL-1ra), and IL-1 type II receptor (IL-1RII), which protect against IL-1β-induced neurotoxicity (101). Administration of the β2-adrenoceptor agonist clenbuterol or the SNRI reboxetine increased the expression of IL-1β, IL-1ra, and IL-1RII in the rat cortex. However, when the rat cortex was activated by systemic LPS, clenbuterol reduced IL-1β expression while maintaining its ability to upregulate IL-1RII and IL-1Ra (98), highlighting the complexity of NE-mediated regulation of the IL-1 system. In addition to IFN-γ and IL-1, NE regulates the NFκB pathway. Pre-treatment with the β_2_-adrenoceptor agonist clenbuterol suppressed LPS-induced NFκB activation, blocked LPS-induced IκBα phosphorylation and degradation, and downregulated the NFκB-inducible genes TNF-α and ICAM-1 in the cortex and hippocampus (102).

### 4.2 Serotonin

Unlike NE, which mainly has anti-inflammatory effects, 5-HT plays a dual role in neuroinflammation, with effects that depend on its concentration, receptor subtypes, and cellular contexts. Under basal conditions, 5-HT can promote inflammation, as shown in SERT knockout models, where increased extracellular 5-HT enhances IL-1β and CD11b mRNA expression, indicating elevated microglial activation (103). However, under pathological or injury conditions, 5-HT exerts anti-inflammatory effects, primarily by enhancing microglial neuroprotective functions and promoting astrocytic release of transforming growth factor β (TGF-β1) (104). Different 5-HT receptor subtypes can mediate distinct effects on inflammation. 5-HT1a activation inhibits NF-κB, reducing proinflammatory cytokine release and mediating neuroprotective effects (104). 5-HT7 has also been shown to promote anti-inflammatory and neuroprotective effects in microglia and astrocytes (105–107). On the other hand, 5-HT2a, 5-HT2b and 5-HT2c are linked to microglial activation and increased neuroinflammation by enhancing NF-κB signaling (26, 32). These studies highlight the complexity of 5-HT-induced regulation of neuroinflammation.

5HT receptors are expressed in both microglia and astrocytes. Acting through these receptors, 5-HT influences microglial polarization, shifting their state between proinflammatory (M1) and anti-inflammatory (M2) phenotypes. Activation of 5-HT1a receptors skews microglia toward the M2 phenotype, reducing TNF-α and IL-1β expression, while 5-HT2 receptors are associated with the M1 state, promoting inflammatory responses (108–110). The 5-HT concentration seems to play a decisive role in microglia activation, as high 5-HT concentrations augment TNF-α release in LPS-stimulated microglia (111, 112). Additionally, 5-HT modulates microglial motility, as demonstrated by Krabbe et al., who found that serotonin enhances microglial movement in response to injury, suggesting a role in neuroprotection and tissue repair (113). Astrocytes expressing 5-HT receptors also contribute to the serotonergic regulation of neuroinflammation (113, 114). Pousset et al. found that low-5-HT levels induce both proinflammatory (IL-6, TNF-α) and anti-inflammatory (TGF-β) cytokine expression in rat hippocampal astrocytes (115), again demonstrating the bidirectional regulation by 5-HT. Intriguingly, SSRIs like paroxetine and fluoxetine, which increase extracellular 5-HT, suppress TNF-α, nitric oxide (NO), and NFκB activity, reducing microglial activation and neutrophil infiltration in inflammatory conditions (111, 116–118). More research is needed to fully elucidate the role of 5-HT in neuroinflammation.

### 4.3 Dopamine

In the CNS, the five DA receptors are differently distributed in brain regions with the relative density: D_1_ > D_2_ > D_3_ > D_5_ > D_4_ (119, 120). DA signaling has been found critical for regulating glial cell activation and neuroinflammation. Both astrocytic and microglial cells have been reported to express DA receptors, which may be altered during inflammation (121, 122). Recent studies have revealed multiple mechanisms by which DA regulates neuroinflammation (123).

Using gain-of-function studies, Shao et al. first demonstrated that the astrocytic DRD2 could suppress neuroinflammation through controlled αB-crystallin (CRYAB), a known mediator of neuroinflammation. Analogously, global knockout of *Drd2*, but not *Drd1* or *Drd3*, pronounced activation of astrocyte and microglia cells, with significant upregulation of proinflammatory mediator genes, including IL-1β, IL-2, IL-6, IL-12β, COX2, and downregulation of the anti-inflammatory cytokine gene, IL-10 (but not TNF-α) (70). In the conditional astrocytic *Drd2*−knockout mouse, while mesencephalic dopaminergic neurons were maintained, levels of proinflammatory mediators in the substantia nigra were remarkably elevated, indicating the critical role of astrocytic DRD2 in neuroinflammation regulation. The authors further found that DRD2 tightly controlled CRYAB expression, required for *Drd2*-mediated suppression of proinflammatory response, in astrocytes. Moreover, administration of the DRD2 agonist quinpirole significantly inhibited 1-methyl-4-phenyl-1,2,3,6-tetrahydropyridine (MPTP)-induced activation of astrocytes and microglia, while also suppressing the expression of proinflammatory mediators, and loss of nigral dopaminergic neurons, in control, but not *Drd2*-null or *Cryab*-null, mice.

Although DRD2 was found highly expressed in neurons, *in vivo* selective neuronal deletion of *Drd2* only mildly increased levels of proinflammatory mediators. Conversely, despite very low DRD2 levels in resident microglia from healthy mouse brains, DRD2 could be induced in microglia upon activation (122, 124). Likewise, Zhang et al. reported upregulated endogenous DRD2 on astrocytes and microglia following intracerebral hemorrhage (ICH)-induced brain injury (124). They further found that *DRD2* knockdown aggravated neurobehavioral deficits, with pronounced expression of cytokines and chemokines (including IL-1β and MCP-1) post-ICH (124). Moreover, the DRD2 agonists, quinpirole and ropinirole, suppressed microglia activation and ameliorated neurobehavioral deficits after ICH, likely mediated by αB-crystallin and enhanced by cytoplasmic binding activity of NF-κB.

DA can also inhibit activation and inflammation of the NLRP3 inflammasome through DRD1 signaling (125), promoting the maturation and release of several proinflammatory cytokines, such as IL-1β and interleukin-18 (IL-18) (126). Yan et al. reported that DA treatment could inhibit IL-1β upregulation by the bacterial neurotoxin nigericin in both microglia and astrocytes, and DA’s inhibitory effects were impaired in *Drd1*-null cells (125). Moreover, *Drd1* knockout mice showed more inflammasome activation, by analyzing IL-1β and IL-18 production or caspase-1 activation, which were impaired in *Drd1* and *Nlrp3* double-knockout mice. They further found higher NLRP3 expression and less NLRP3 ubiquitination following MPTP neurotoxin treatment (a model for PD) in brains from *Drd1*-knockout versus control mice (126). Taken together, these results suggest that DRD1 signaling prevents neuroinflammation by inhibiting the NLRP3 inflammasome by promoting NLRP3 ubiquitination. Likewise, Wang et al. reported that DRD1 activation decreased NLRP3-mediated inflammation in ICH-induced mice (127). A DRD1-specific agonist, A68930, inhibited microglia activation, neutrophil infiltration, and expression of NLRP3, caspase 1, and IL-1β, in ICH mice. In contrast to the inhibitory role of DRD1 signaling in neuroinflammation, Wang et al. recently reported that methamphetamine exacerbates an LPS pro-inflammatory response by activating D_1_-like receptors (128), suggesting complex regulation of neuroinflammation by DA.

Although there is a relatively low expression in the CNS, D3R (DRD3) displays the highest selectivity for DA (Ki ≈ 27 nM), followed by D5R (Ki ≈ 228 nM) and then D4R, D2R, and D1R (129–132). While DRD3 is strongly involved in peripheral and central inflammation, in several experimental systems (133) its role in neuroinflammation regulation remains controversial. For example, Elgueta et al. showed that the D3R-selective antagonist PG01037, when administered intraperitoneally, improves locomotor performance, reduces dopaminergic neuron loss in the nigrostriatal pathway, and promotes astrogliosis and microglial ramification in an MPTP-induced PD mouse model. This study further demonstrates that D3R antagonism protects dopaminergic neurons, enhances motor function, and modulates neuroinflammation by reducing astrocyte reactivity, which may subsequently activate an anti-inflammatory signal to microglia (134). In line with this, IFN-γ, a critical cytokine in stimulating and maintaining glial cell activation, was upregulated in wild-type CD4^+^ T cells, following DRD3 agonism by PD128907, but decreased by DRD3 deficiency under Th1 differentiation conditions. In contrast, DRD3 deficiency resulted in chronic depression as measured by increased immobility (135–137) and neuroinflammation, as evidenced by increased activation of microglia but not astrocytes, as well as elevated mRNA expression of the proinflammatory cytokines TNF-α, IL-1β, and IL-6, in mesolimbic dopaminergic regions (136). Importantly, microglial inhibition partially ameliorated depressive-like behavior and neuroinflammation in selected mesolimbic reward areas induced by D3R deficiency.

The discrepancy described above could be due to DRD3 cell-specific effects on pro- or anti-inflammatory responses. In Elgueta’s study, CD4^+^ T cells infiltrated into the CNS after MPTP treatment (134), while low levels of DA selectively activated DRD3 (due to its high affinity), in CD4+ T cells, which preferably differentiated into proinflammatory Th1 cells. Subsequently, IFN-γ and TNF-α, secreted by Th1 CD4^+^ T cells, enhanced MPTP-induced microglia activation, contributing to neuroinflammation (134). Conversely, in Wang’s studies, microglial DRD3 signaling was proinflammatory, under basal conditions (138). Analogously, DRD3 deficiency resulted in microglia activation and subsequently, chronic inflammation (138).

## 5 Neuroinflammation effects on monoamine signaling

**Figure 1:**
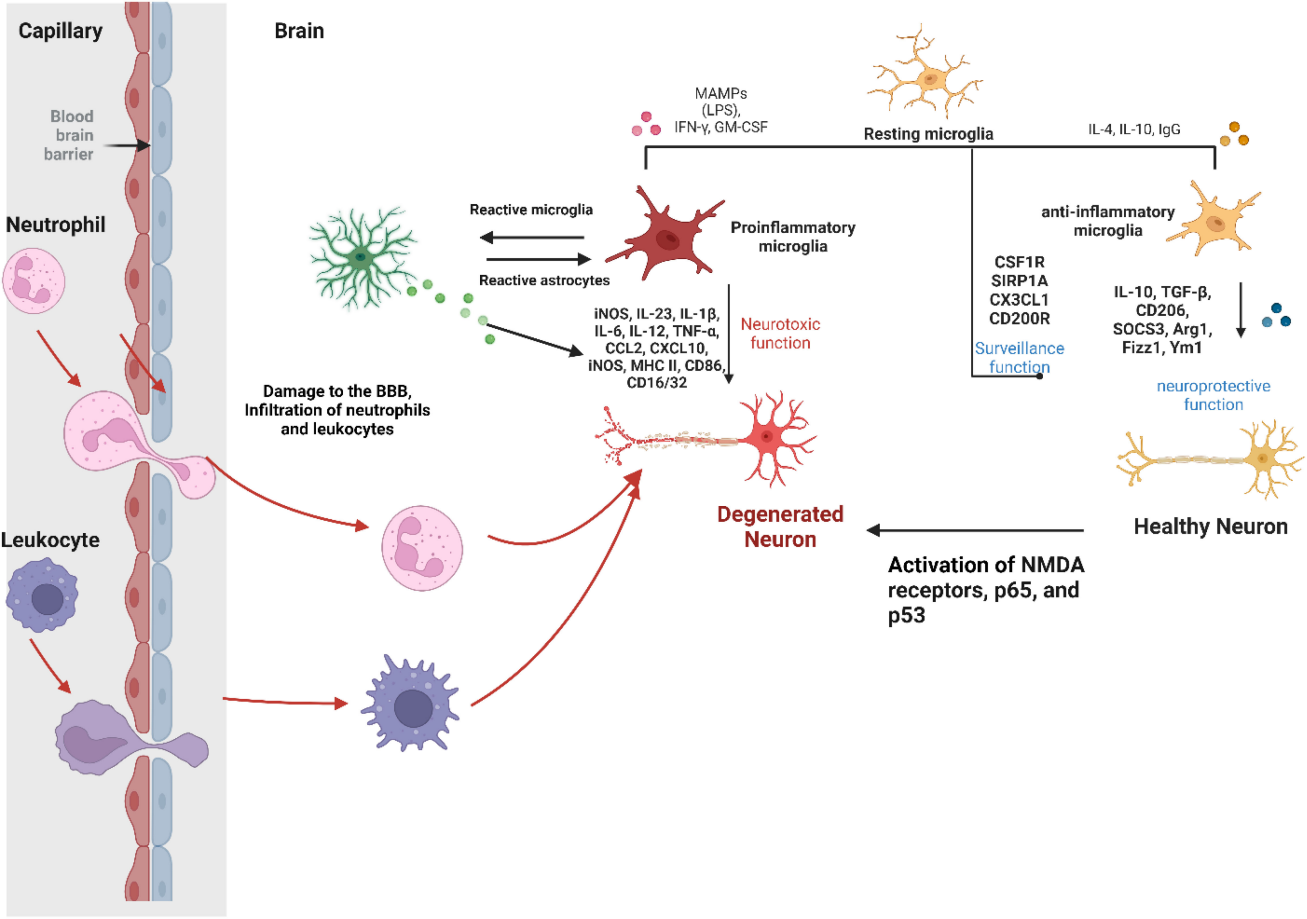
Role of microglia and astrocytes in brain neuroinflammation. Neuroinflammation in the brain, and the role of microglia. Left, damage to the blood-brain barrier (BBB) allows neutrophils and leukocytes into the brain, activating microglia. The central section illustrates microglia in different states: resting, proinflammatory, and anti-inflammatory, withproinflammatory microglia releasing harmful mediators, and anti-inflammatory microglia being protective. Reactive astrocytes interact with activated microglia, affecting inflammatory response. Right, the image contrasts a healthy neuron with a degenerated one, emphasizing how inflammation and receptor activation affect neuronal damage and neurological disorders. Created with Biorender.com.

Various stimuli, including viral and bacterial infections, can disrupt brain neurochemistry, leading to impaired neurotransmission. More specifically, evidence suggests close communication between the immune system and the CNS, as mediated by an immune-neural-synaptic axis. This involves peripheral immune cells infiltrating the brain or activation of glial cells via humoral immune factors that cross the BBB (**Figure 1**) (36, 37). During neuroinflammation, pro- and anti-inflammatory cytokines, synthesized and released from activated local glial cells and infiltrating immune cells, can in turn regulate monoamine signaling by affecting their synthesis, release, reuptake, and turnover (139–144). Cytokines can also participate in neurogenesis, or mediate cell death of neurons in the CNS, to indirectly affect monoamine synthesis (82, 145).

### 5.1 Cytokine effects on monoamine synthesis

There are at least two major pathways by which cytokines regulate the synthesis of monoamine neurotransmitters. First, proinflammatory cytokines, including IL-6, IFN‐γ, IL‐1, and TNF-α, may reduce 5-HT synthesis by reducing the availability of its precursor, tryptophan (146–150), via its catabolism into kynurenine by indoleamine-2,3-dioxygenase (IDO), an enzyme present in glial cells and neurons (151). Second, proinflammatory cytokines may interfere with DA synthesis by acting on tetrahydrobiopterin (BH4) (152), a cofactor of three aromatic amino acid hydroxylase enzymes (153) that degrades the amino acid phenylalanine, which is used in the biosynthesis of the neurotransmitters 5-HT, NE, DA, and EPI (154). Although proinflammatory cytokines, including TNFα and IL-1β, can induce BH4 production, they can also stimulate NOS production of NO, a reaction that requires BH4 as a cofactor, decreasing the availability of BH4 for monoamine synthesis (155–157). Proinflammatory cytokines, such as IFN-γ, can also trigger high output of ROS, which can destroy the oxidation-labile BH4, reducing monoamine biosynthesis (158).

### 5.2 Cytokine effects on monoamine release

Proinflammatory cytokines can also influence monoamine signaling by affecting neurotransmitter release. For instance, it was reported that IL-1α could stimulate DA release by activating type II protein kinase A in PC-12 cells (159), while IL-1β injected directly into the rat anterior hypothalamus, elicited the release of NE, DA, and 5-HT, also increasing their metabolites (160). Moreover, administration of IFN-α to Hepatitis C patients increased reuptake and decreased release of radiolabeled DOPA, the primary precursor of DA (161). This was possibly related to increased cranial production of the tryptophan metabolite kynurenine acid (KA), via activation of IDO, as intrastriatal KA administration (by *in vivo* micro-dialysis) is known to decrease extracellular DA, in rats (162).

### 5.3 Cytokine effects on monoamine reuptake

Monoamine transporters (MATs) regulate neurotransmission via reuptake of DA, 5-HT, and NE from extra-neuronal regions and decrease the synaptic availability of monoamine neurotransmitters. There are three main MAT members: the DA transporter (DAT), SERT, and the NE transporter (163–169). Several proinflammatory cytokines, such as IL-1β, TNF-α, IFN-α, and IFN-γ, can upregulate SERT activity (thus reducing availability of extracellular 5-HT) (170–172) while the anti-inflammatory cytokine IL-4 can inhibit SERT activity (173). Similarly, Wu et al. found increased DAT levels following injection of the proinflammatory cytokine TNF-α (174). Many signaling pathways regulate MATs, including mitogen‐activated protein kinase (MAPK) (175) which is activated by increased levels of proinflammatory cytokines such as IL‐1β and TNF‐α, consequently enhancing cell membrane transporter activity and the reuptake of the neurotransmitters 5‐HT, DA, and NE (140, 176, 177).

### 5.4 Cytokine effects on monoamine turnover

Proinflammatory cytokines may also regulate monoamine signaling through neurotransmitter turnover. For instance, intraperitoneal IL-1 injection increased NE turnover in the whole hypothalamus and several specific hypothalamic nuclei, but not in the medulla oblongata and cerebral cortex (178), while systemic administration of TNF-α into CD-1 mice altered central NE, DA, and 5-HT turnover (179). Likewise, intracerebroventricular infusion of IFN-α into the rat brain promoted 5-HT and DA turnover in the prefrontal cortex and CA2/CA3 hippocampal regions, respectively, as suggested by findings of increased ratios of major metabolites and parental amines (180). A recent study also revealed that significant dopaminergic turnover within the dorsal striatum was induced by IFN-γ treatment (181). Specifically, the effects of proinflammatory cytokines on monoamine turnover largely depend on the specific cytokine, treatment duration, and specific brain region (182).

## 6 Interconnection of monoamine signaling and neuroinflammation in neuropsychiatric disorders

Research into neuroinflammation and its connection to psychiatric disorders is expanding quickly. A better understanding of this relationship could revolutionize how we treat mental illnesses. This could lead to more targeted and effective therapies that alleviate symptoms and address the underlying causes of these disorders. Classical psychiatric disorders (e.g., schizophrenia, autism, major depression, bipolar disorder, and obsessive-compulsive disorder) and neurological diseases with psychiatric symptoms (e.g. AD and PD) are widely associated with neuroinflammation and immunological abnormalities, in addition to disrupted neurotransmission. Many of these disorders exhibit both chronic neuroinflammation and dysregulated monoamine signaling, suggesting potential interconnections (9, 82, 144). This section discusses the interconnection between each monoamine system and neuroinflammation and how these processes contribute to neurological diseases, particularly MDD, AD, and PD.

### 6.1 Monoamine signaling and neuroinflammation in major depressive disorder

MDD is a complex and heterogeneous mental health condition characterized by persistent low mood, anhedonia, and cognitive impairments. Its pathophysiology involves disruptions in multiple neurobiological systems, including monoamine neurotransmission and neuroinflammation. A growing body of evidence suggests that neuroinflammation not only contributes to MDD but also interacts with MA-ergic pathways, exacerbating neurotransmitter imbalances and influencing treatment response (183–187). Elevated inflammation in both PNS and CNS, along with glial cell activation, has been consistently linked to MDD (188, 189), highlighting inflammation as a key contributor to disease pathology. Studies suggest that IL-1R/C3/C3aR activation in astrocytes and microglia may contribute to abnormal synaptic pruning in depression, supporting the inflammatory hypothesis of MDD (190). Pro-inflammatory cytokines such as TNF-α, IL-6, and CRP, are consistently elevated in MDD patients (183, 184, 186, 187). These pro-inflammatory cytokines can cross the BBB, influencing brain regions like the insula, which governs emotional processing and cognition (191, 192). Elevated cytokine levels correlate with reduced connectivity in these regions, leading to depressive rumination (193).

Chronic stress, a major environmental risk factor, induces neuroinflammatory processes that disrupt monoamine neurotransmission and modulate monoamine metabolism, reuptake, and receptor function, contributing to imbalances in 5-HT, NE, and DA (194–196). Moreover, chronic stress enhances the kynurenine pathway, leading to higher oxidative stress and neurotoxicity, which in turn disrupts the balance of monoamines and impairs neuronal health (196). In addition to stress, obesity, metabolic dysfunction, genetics, and lifestyle also contribute to inflammation and increase the risk for psychiatric illness (197).

DA, essential for reward processing and motivation, is implicated in anhedonia, a core symptom of MDD (198). Laboratory studies have demonstrated that inflammatory stimuli affect neurotransmitters and reward-processing circuits, including the ventral striatum and ventromedial prefrontal cortex, which are associated with reduced motivation (197). Neuroimaging studies also confirm that increased endogenous inflammation is associated with decreased activation and reduced functional connectivity within reward circuits, reinforcing the association between inflammation and anhedonia (197). Inflammation’s impact on neurotransmitter synthesis, release, and reuptake, particularly of DA and glutamate, appears to drive motivational deficits. For example, inflammation-induced DA dysfunction has been linked to elevated CRP levels, which correlate with reduced DA synthesis and release (197). Chronic stress-induced inflammation further disrupts DA signaling, particularly in the VTA and nucleus accumbens, regions involved in reward processing. These studies collectively suggest that inflammation as a critical factor influencing brain circuits responsible for reward and motivation (197).

In addition to DA signaling, recent findings highlight that inflammation modulates 5-HT signaling (199, 200). Inflammation activates the indoleamine 2,3-dioxygenase (IDO) enzyme, which shifts tryptophan metabolism away from 5-HT production toward the neurotoxic kynurenine pathway (194, 195). This results in 5-HT depletion and increased neurotoxic metabolites, exacerbating depressive symptoms. Inflammation also depletes tryptophan, impairing 5-HT production (201). On the other hand, 5-HT regulates immune responses, and dysregulation of this interaction has been implicated in MDD, particularly in patients with elevated inflammatory markers (200). Genetic variations in the SERT can influence immune system activity, further supporting a bidirectional relationship between 5-HT signaling and neuroinflammation (196).

Given the interplay between inflammation and neurotransmitter systems, it is not surprising that antidepressant responses vary depending on an individual’s inflammatory status. SSRIs increase 5-HT availability but may be less effective in individuals with high inflammation, as cytokines impair 5-HT receptor function (199). In contrast, dopaminergic agents (e.g., bupropion, L-DOPA) may be more effective in patients with elevated CRP or IL-17 levels, as they target inflammation-related DA deficits (200) Indeed, MDD patients with CRP >2 mg/L show increased ventral striatum-ventromedial prefrontal cortex connectivity following L-DOPA administration, suggesting that DA-enhancing treatments may be particularly beneficial in inflammation-associated depression (197). Additionally, elevated IL-17 levels have been identified as a biomarker for antidepressant response, with patients showing better responses to dopaminergic antidepressants like bupropion than to SSRIs alone (200, 202, 203). By contrast, patients with low IL-17 levels respond better to SSRI monotherapy (200). Additionally, TNF-α inhibitors have demonstrated antidepressant effects in high-inflammation patients (204, 205), though some evidence suggests that their combination with SSRIs may reduce efficacy (189, 206). These studies underscore the need for precision medicine in antidepressant selection.

The recognition of both monoamine dysfunction and neuroinflammation has led to the development of treatments targeting these mechanisms. SSRIs and SNRIs aim to increase monoamine availability, and they also support neuroplasticity by increasing brain-derived neurotrophic factor (BDNF) and exhibit modest anti-inflammatory effects, suggesting a connection between inflammation and MDD (207, 208). However, their efficacy can diminish over time due to SERT degradation, particularly when inflammation is involved (209). Some medications can reduce proinflammatory cytokines like TNF-α and IL-6, further emphasizing the role of inflammation in MDD. Combining antidepressants with anti-inflammatory treatments may enhance therapeutic outcomes (210).

Alongside traditional treatments, alternative pharmacological therapies have shown promise. Ketamine, for example, targets NMDA receptors, promotes neuroplasticity, reduces inflammation, and provides rapid relief from suicidal ideation and treatment-resistant depression (211, 212). Cannabinoids, such as THC and CBD, offer both antidepressant and anti-inflammatory effects, with CBD also providing neuroprotective benefits. Psychedelics like psilocybin and ayahuasca, which target the 5-HT2A receptor, have shown potential in reducing neuroinflammation and improving mood, offering long-lasting improvements in mood regulation. However, further research is required to fully understand their mechanisms and long-term effectiveness (212).

The mechanistic links between monoamine signaling and neuroinflammation provide critical insights into the pathophysiology of MDD. Inflammation disrupts 5-HT and DA systems, contributing to treatment resistance in some patients. Identifying biomarkers such as CRP and IL-17 may help guide personalized treatment strategies, optimizing the use of monoaminergic antidepressants, dopaminergic agents, and anti-inflammatory therapies. Future research should focus on refining precision medicine approaches to improve treatment outcomes for individuals with inflammation-associated depression.

### 6.2 Monoamine signaling and neuroinflammation in Alzheimer’s disease

In AD, pathology often begins with disruptions in monoamine systems: dopaminergic, noradrenergic, and serotonergic signaling (213). Early tau abnormalities target key regions like the locus coeruleus and serotonergic nuclei, initiating the spread of disease to the hippocampus and neocortex. These monoaminergic neurons, with their long, unmyelinated axons and high metabolic demands, are particularly vulnerable to degeneration. This early loss contributes to cognitive decline, neuropsychiatric symptoms (like depression and agitation), and motor deficits (4, 213). For instance, the locus coeruleus, a hub for NE, suffers significant neuronal loss, leading to altered NE transmission and impaired attention and memory (214). Surviving neurons attempt to compensate by sprouting dendrites, ramping up synthesis, and slowing reuptake, which elevates cerebrospinal fluid NE levels (215, 216). This adaptation may cause heightened NE activity via α_2A_ (217, 218), and β2 (219, 220) adrenergic receptors to promote amyloid-beta (Aβ) deposition. Similarly, 5-HT deficits impair cognition and mood regulation, while DA loss affects both motor function and executive abilities. Studies show reduced nucleolar volume and RNA in these neurons, underscoring the profound impact of their dysfunction (213).

As AD progresses, neuroinflammation takes the center stage, driven by microglial activation. Aβ plaques trigger microglia through receptors like toll-like receptors (TLRs) and RAGE, prompting the release of pro-inflammatory cytokines such as TNF-α and IL-1β (10, 164). These cytokines exacerbate neurodegeneration and directly disrupt monoamine signaling (221). Activated microglia increase indoleamine 2,3-dioxygenase (IDO), diverting tryptophan from 5-HT synthesis to the kynurenine pathway, thus depleting 5-HT (222). Inflammation also activates p38 MAPK, accelerating the clearance of DA and NE, further deepening cognitive and mood disturbances (223–225).

Microglial activation is not only involved in neurodegeneration but also plays a critical role in neuropsychiatric symptoms common in AD, including depression, anxiety, agitation, and apathy (226). In regions like the anterior cingulate cortex (part of the default mode network), microglial activation correlates with irritability and negative affect, while elevated cytokine levels track with symptom severity (227). Meanwhile, microglia surrounding Aβ plaques become dysfunctional: they’re meant to phagocytize amyloid, but instead, they fail, perpetuating a cycle of protein accumulation, inflammation, and neurotoxic factor release—ROS, NO, and more. This cascade drives neurodegeneration (228). The kynurenine pathway adds another layer (223), activated microglia convert kynurenine into quinolinic acid, an NMDA receptor agonist. Coupled with impaired glutamate reuptake, this leads to excitotoxicity, reducing BDNF and worsening depressive symptoms—a pattern seen not just in AD but also in diseases like Parkinson’s (229, 230). Some research, like studies in APPswe/PS1ΔE9 mice, suggests that rising cytokines precede drops in SERT activity, with amyloid status further influencing 5-HT function (231).

The interplay among monoamine dysfunction, neuroinflammation, and Aβ accumulation creates a vicious cycle: monoamine deficits fuel early symptoms, neuroinflammation amplifies the damage, and Aβ accumulation keeps the fire burning. Interestingly, disruptions in the default mode network link inflammation to amyloid pathology, suggesting a feedback loop. Some researchers now argue that neuroinflammation may outpace amyloid plaques and tau tangles (232) as a driver of AD progression, with chronic microglial activation accelerating the disease (10, 233). Therapeutically, this opens doors. Targeting microglial activation could break the cycle—post-mortem studies of AD patients treated with an Aβ42 vaccine show reduced microglial activity and plaque burden (234). Recent studies even propose microglial activation as an early diagnostic biomarker, offering a chance to intervene before damage escalates (235, 236). In animal models, deleting receptors like TLR4, TLR6, or CD36 curbs cytokine production and amyloid accumulation, hinting at ways to slow the progression (237–239). In addition, other treatments for neuroinflammation have been explored for AD (240). While not FDA-approved for neurodegenerative diseases, drugs like minocycline, curcumin, vitamin E, and celecoxib, approved for other conditions, target neuroinflammation and may potentially slow disease progression. Pioglitazone, a PPARγ agonist, has shown promise in shifting microglia to a more protective state, which may enhance the clearance of Aβ plaques (240).

Addressing monoamine dysfunction also holds promise: inhibiting IDO could restore 5-HT levels while stabilizing NE might ease cognitive and mood symptoms. Several drugs targeting monoamines and neuroinflammation are being explored in AD (241). Recently, aducanumab (Aduhelm) was approved by the FDA for early-stage AD and mild cognitive impairment, showing promise in reducing amyloid-beta (Aβ) accumulation and halting cognitive decline (4, 241). Lecanemab also demonstrated reduced amyloid markers and slowed cognitive decline in early AD patients. Immunotherapies, mainly targeting tau, are a growing area of research. Some anti-tau vaccines and monoclonal antibodies, like AADvac1, gosuranemab, and tilavonemab, have reached Phase II trials. These treatments utilize the immune system to target Aβ and tau, but their effectiveness and safety are still being evaluated (242). Despite these advances, challenges remain, including recruitment difficulties and high costs of clinical trials. While Aducanumab and Lecanemab provide hope, more targeted therapies are required to address the complex mechanisms of Aβ accumulation, tau aggregation, and neuroinflammation.

### 6.3 Monoamine signaling and neuroinflammation in Parkinson’s disease

Parkinson’s disease is primarily characterized by the loss of dopaminergic neurons in the substantia nigra, resulting in reduced DA levels and motor symptoms such as tremors, rigidity, and bradykinesia. This neurodegeneration is driven by a complex interplay of genetic, environmental, and immunological factors, contributing to the disease’s multifaceted nature, which shares similarities with autoimmune diseases (243). The pathological mechanisms behind PD are similar to those seen in other neurodegenerative disorders and involve neuron dysfunction, protein aggregation, oxidative stress, mitochondrial dysfunction, and neuroinflammation (244).

Microglia, the resident immune cells of the brain, become activated by the presence of misfolded α-synuclein fibrils, which are a hallmark of PD pathology. While these activated microglia attempt to clear the misfolded proteins, they become chronically activated without success. As dopaminergic neurons die, they release pro-inflammatory mediators such as α-synuclein, MMP-3, and ATP, which further activate microglia and contribute to the release of toxic substances that damage neighboring cells (245). Additionally, pro-inflammatory signals from astrocytes intensify microglial activation, while misfolded proteins drive microglial M1 polarization, which is associated with further neurotoxicity. Studies involving animal models, such as chronic MPTP exposure, show a reduction in the protective M2 phenotype of microglia, which can be reversed by using a mixture of M1 and M2-conditioned media, offering potential therapeutic insights (246).

DA signaling itself plays a significant role in regulating neuroinflammation (247). In PD patients, elevated proinflammatory cytokines, such as IL-1β, TNF-α, and IL-6, have been found in the brain in correlation with dopaminergic neuron loss (248–250). Experimental models, such as the MPTP-induced mouse model, also show a strong association between low DA levels and increased neuroinflammation, including microglial activation and T-cell infiltration. The activated microglia release harmful molecules such as TNFα, INF-γ, ROS, and NO, further contributing to neuronal damage (250–252). Notably, enhancing 5-HT or DA signaling through treatments like the 5-HT1A receptor agonist 8-OH-DPAT and levodopa has been shown to reduce neuroinflammation and alleviate symptoms (253, 254).

Given the critical role of inflammation in PD, immunomodulatory therapies have emerged as a promising avenue for treatment. The inhibition of neuroinflammation has proven to be neuroprotective in various studies, including the use of dominant-negative TNF inhibitors to block soluble TNF signaling, which reduces dopaminergic neuron loss in animal models (255). Anti-inflammatory compounds have also shown promise in restoring DA levels and reversing dopaminergic neuron degeneration in PD models (256). Although nonsteroidal anti-inflammatory drugs (NSAIDs) have yielded inconsistent results in epidemiological studies, likely due to methodological differences (257), targeting glial activation and cytokine shows promise. Drugs like minocycline and dexamethasone, which modulate glial activity, have shown potential benefits, while the NLRP3 inhibitor MCC950 has been demonstrated to reduce microglial activation and alleviate PD symptoms (258). In addition, targeting pro-inflammatory cytokines such as IL-1 and TNF using neutralizing antibodies or activating the GLP1 receptor has demonstrated neuroprotective effects, with clinical trials exploring the use of GLP1 analogs like semaglutide (NCT03659682). Immunomodulation of the peripheral immune system, such as T-cell transfer, has also demonstrated protective effects on dopaminergic neurons (259). Furthermore, immunomodulatory approaches aiming to resolve inflammation, such as the use of resolvin D1, have also been shown to alleviate motor deficits in PD models (260).

While current PD treatments primarily focus on symptom management, typically through maintaining DA levels with inhibitors (monoamine oxidase B and catechol-O-methyl transferase) or DA precursors like levodopa, these therapies do not modify the underlying disease (261). Immunotherapy targeting α-synuclein aggregation, either through active immunization or passive antibody administration, is currently under investigation in clinical trials (262). Research continues to explore potential disease-modifying approaches, with particular attention to how inflammation, neuroinflammation, and monoamine signaling contribute to PD pathophysiology. The complex relationship between DA signaling and neuroinflammation in PD offers opportunities for novel therapeutic strategies. Targeting neuroinflammation, whether through cytokine modulation, glial activation, or α-synuclein aggregation inhibition, holds promise for slowing or halting disease progression. However, more research is needed to clarify the exact causative factors and to identify effective disease-modifying treatments for PD.

## 7 Conclusion and perspectives

The bidirectional relationship between monoamine signaling and neuroinflammation has emerged as a pivotal factor in the pathophysiology of various neuropsychiatric and neurodegenerative disorders. This interplay, marked by the mutual influence of monoamine neurotransmitters (NE, 5-HT, and DA) and inflammatory processes, is central to understanding diseases like MDD, AD, and PD. Chronic dysregulation of monoamine signaling leads to detrimental consequences for brain function and behavior. Monoamine neurotransmitters also affect immune and glial cells, regulating their migration, proliferation, and inflammasome activation and altering cytokine secretion and various neuroinflammatory processes. Conversely, neuroinflammation disrupts monoamine function by modifying neurotransmitter synthesis, release, reuptake, and turnover, creating a feedback loop that exacerbates the progression of neuropsychiatric and neurodegenerative diseases. Therefore, therapeutic approaches aimed at both normalizing monoamine levels and modulating neuroinflammatory processes offer promising new directions for treatment.

For MDD, the therapeutic landscape is evolving with a focus on combining traditional antidepressants, which primarily target monoamine systems, with anti-inflammatory strategies. Although SSRIs and SNRIs remain the cornerstone of MDD treatment, their anti-inflammatory properties are gaining attention, particularly in treatment-resistant cases. The combination of NSAIDs, cytokine inhibitors, or biologics with conventional antidepressants holds promise in mitigating the neuroinflammation that often accompanies depression (263). In AD, the complex interplay between monoaminergic degeneration, neuroinflammation, Aβ plaque accumulation, and tau pathology requires innovative cocktail therapies. Targeting the monoamine system and neuroinflammation would not only help control neuropsychiatric symptoms, which are prevalent in AD (4), but also hold the potential to slow disease progression. New clinical approaches may combine these approaches with immunotherapies that target Aβ, potentially slowing the progression of the disease and improving patient outcomes. In addition to pharmacological treatments, non-pharmacological interventions such as exercise, dietary modifications, and mindfulness-based therapies are gaining recognition for their ability to modulate neuroinflammatory pathways, potentially enhancing treatment outcomes across all three disorders (264–266). In PD, preclinical studies have highlighted the potential of combining anti-inflammatory treatments with dopaminergic therapies, with NSAIDs, alpha-lipoic acid, and other immunomodulatory agents showing promise in protecting dopaminergic neurons (267). Future therapies will likely focus on neuroprotection while simultaneously addressing inflammation, potentially offering more comprehensive treatment options for PD.

Given the complexity of monoamine signaling and neuroinflammation, advancing therapeutic strategies for diseases involving these systems requires the use of cutting-edge research techniques. Emerging methodologies, such as single-cell RNA sequencing, spatial transcriptomics, and 3D *in vitro* models, provide unprecedented cellular and molecular insights into the dynamics of glial activation, neurotransmitter signaling, and inflammatory pathways, facilitating the identification of novel therapeutic targets. New organ-on-chip systems and animal models that closely replicate human disease states are available for the preclinical evaluation of novel therapies (268). Imaging techniques like positron emission tomography and magnetic resonance imaging allow monitoring neuroinflammation and assessing the efficacy of treatments *in vivo* (269). With the advancement of new technologies, researchers are better equipped to address monoamine dysfunction and neuroinflammation for improved treatment strategies.

In conclusion, integrating anti-inflammatory strategies with traditional treatments targeting monoamine dysfunction holds significant promise for improving the management of MDD, AD, and PD. Addressing both systems simultaneously could lead to more effective, holistic therapies that alleviate symptoms, modify disease progression, and enhance overall functional outcomes.

## References

[1] Ng J Papandreou A Heales SJ Kurian MA. Monoamine neurotransmitter disorders–clinical advances and future perspectives. Nat Rev Neurol. (2015) 11:567–84. doi: 10.1038/nrneurol.2015.172 26392380

[2] Jiang Y Zou D Li Y Gu S Dong J Ma X. Monoamine neurotransmitters control basic emotions and affect major depressive disorders. Pharm (Basel). (2022) 15(10):1203. doi: 10.3390/ph15101203 PMC9611768 36297314

[3] Doummar D Moussa F Nougues MC Ravelli C Louha M Whalen S. Monoamine neurotransmitters and movement disorders in children and adults. Rev Neurol (Paris). (2018) 174:581–8. doi: 10.1016/j.neurol.2018.07.002 30166070

[4] Saggu S Bai A Aida M Rehman H Pless A Ware D. Monoamine alterations in Alzheimer’s disease and their implications in comorbid neuropsychiatric symptoms. Geroscience. (2025) 47(1):457–82. doi: 10.1007/s11357-024-01359-x PMC11872848 39331291

[5] Wu H Denna TH Storkersen JN Gerriets VA. Beyond a neurotransmitter: The role of serotonin in inflammation and immunity. Pharmacol Res. (2019) 140:100–14. doi: 10.1016/j.phrs.2018.06.015 29953943

[6] Subhramanyam CS Wang C Hu Q Dheen ST. Microglia-mediated neuroinflammation in neurodegenerative diseases. Semin Cell Dev Biol. (2019) 94:112–20. doi: 10.1016/j.semcdb.2019.05.004 31077796

[7] Gilhus NE Deuschl G. Neuroinflammation - a common thread in neurological disorders. Nat Rev Neurol. (2019) 15:429–30. doi: 10.1038/s41582-019-0227-8 31263256

[8] Carthy E Ellender T. Histamine, neuroinflammation and neurodevelopment: A review. Front Neurosci. (2021) 15. doi: 10.3389/fnins.2021.680214 PMC8317266 34335160

[9] Wang Q Liu Y Zhou J. Neuroinflammation in Parkinson’s disease and its potential as therapeutic target. Transl Neurodegener. (2015) 4:19. doi: 10.1186/s40035-015-0042-0 26464797 PMC4603346

[10] Heneka MT Carson MJ El Khoury J Landreth GE Brosseron F Feinstein DL. Neuroinflammation in alzheimer’s disease. Lancet Neurol. (2015) 14(4):388–405. doi: 10.1016/S1474-4422(15)70016-5 25792098 PMC5909703

[11] Troubat R Barone P Leman S Desmidt T Cressant A Atanasova B. Neuroinflammation and depression: A review. Eur J Neurosci. (2021) 53:151–71. doi: 10.1111/ejn.14720 32150310

[12] Duman RS Aghajanian GK Sanacora G Krystal JH. Synaptic plasticity and depression: new insights from stress and rapid-acting antidepressants. Nat Med. (2016) 22:238–49. doi: 10.1038/nm.4050 PMC5405628 26937618

[13] Jesulola E Micalos P Baguley IJ. Understanding the pathophysiology of depression: From monoamines to the neurogenesis hypothesis model-are we there yet? Behav Brain Res. (2018) 341:79–90. doi: 10.1016/j.bbr.2017.12.025 29284108

[14] Rosenbaum DM Rasmussen SG Kobilka BK. The structure and function of G-protein-coupled receptors. Nature. (2009) 459:356–63. doi: 10.1038/nature08144 PMC3967846 19458711

[15] Sun L Ye RD. Role of G protein-coupled receptors in inflammation. Acta Pharmacol Sin. (2012) 33:342–50. doi: 10.1038/aps.2011.200 PMC4085652 22367283

[16] Ge YJ Liao QW Xu YC Zhao Q Wu BL Ye RD. Anti-inflammatory signaling through G protein-coupled receptors. Acta Pharmacol Sin. (2020) 41:1531–8. doi: 10.1038/s41401-020-00523-1 PMC7921558 33060777

[17] Zou H-L Li J Zhou J-L Yi X Cao S. Effects of norepinephrine on microglial neuroinflammation and neuropathic pain. Ibrain. (2021) 7:309–17. doi: 10.1002/ibra.12001 PMC10528971 37786561

[18] Giorgi FS Saccaro LF Galgani A Busceti CL Biagioni F Frati A. The role of Locus Coeruleus in neuroinflammation occurring in Alzheimer’s disease. Brain Res Bull. (2019) 153:47–58. doi: 10.1016/j.brainresbull.2019.08.007 31419539

[19] Kandel ER Schwartz JH Jessell TM. Principles of Neural Science. 4th ed. New York: McGraw-Hill, Health Professions Division (2000).

[20] Theibert AB. Neurotransmitter systems II: monoamines, purines, neuropeptides, & Unconventional neurotransmitters. In: Amthor FR Theibert AB Standaert DG Roberson ED, editors. Essentials of Modern Neuroscience. McGraw Hill, New York, NY (2020).

[21] Racké K Reimann A Schwörer H Kilbinger H. Regulation of 5-HT release from enterochromaffin cells. Behav Brain Res. (1996) 73(1-2):83–7. doi: 10.1016/0166-4328(96)00075-7 8788482

[22] Hornung JP. The human raphe nuclei and the serotonergic system. J Chem Neuroanat. (2003) 26:331–43. doi: 10.1016/j.jchemneu.2003.10.002 14729135

[23] Jacobs BL Azmitia EC. Structure and function of the brain serotonin system. Physiol Rev. (1992) 72:165–229. doi: 10.1152/physrev.1992.72.1.165 1731370

[24] Berger M Gray JA Roth BL. The expanded biology of serotonin. Annu Rev Med. (2009) 60:355–66. doi: 10.1146/annurev.med.60.042307.110802 PMC5864293 19630576

[25] Mohammad-Zadeh L Moses L Gwaltney-Brant S. Serotonin: a review. J Vet Pharmacol Ther. (2008) 31:187–99. doi: 10.1111/j.1365-2885.2008.00944.x 18471139

[26] Shajib MS Khan WI. The role of serotonin and its receptors in activation of immune responses and inflammation. Acta Physiol. (2015) 213:561–74. doi: 10.1111/apha.2015.213.issue-3 25439045

[27] Brenner B Harney JT Ahmed BA Jeffus BC Unal R Mehta JL. Plasma serotonin levels and the platelet serotonin transporter. J Neurochem. (2007) 102:206–15. doi: 10.1111/j.1471-4159.2007.04542.x PMC3041643 17506858

[28] Prah A Purg M Stare J Vianello R Mavri J. How monoamine oxidase A decomposes serotonin: an empirical valence bond simulation of the reactive step. J Phys Chem B. (2020) 124:8259–65. doi: 10.1021/acs.jpcb.0c06502 PMC7520887 32845149

[29] Chu A Wadhwa R. Selective Serotonin Reuptake Inhibitors. StatPearls. Treasure Island (FL: StatPearls Publishing Copyright © (2023). StatPearls Publishing LLC.; 2023. 32119293

[30] Kishore S Stamm S. The snoRNA HBII-52 regulates alternative splicing of the serotonin receptor 2C. science. (2006) 311:230–2. doi: 10.1126/science.1118265 16357227

[31] D’Souza UM Craig IW. Functional polymorphisms in dopamine and serotonin pathway genes. Hum Mutation. (2006) 27:1–13. doi: 10.1002/(ISSN)1098-1004 16320307

[32] Herr N Bode C Duerschmied D. The effects of serotonin in immune cells. Front Cardiovasc Med. (2017) 4:48. doi: 10.3389/fcvm.2017.00048 28775986 PMC5517399

[33] Speranza L di Porzio U Viggiano D de Donato A Volpicelli F. Dopamine: the neuromodulator of long-term synaptic plasticity, reward and movement control. Cells. (2021) 10(4):735. doi: 10.3390/cells10040735 33810328 PMC8066851

[34] Jaber M Robinson SW Missale C Caron MG. Dopamine receptors and brain function. Neuropharmacology. (1996) 35:1503–19. doi: 10.1016/S0028-3908(96)00100-1 9025098

[35] Lang AE Lozano AM. Parkinson’s disease. New Engl J Med. (1998) 339:1130–43. doi: 10.1056/NEJM199810153391607 9770561

[36] Koob GF Bloom FE. Cellular and molecular mechanisms of drug dependence. Science. (1988) 242:715–23. doi: 10.1126/science.2903550 2903550

[37] Feldman R Limbird L Nadeau J Fitzgerald G Robertson D Wood A. Dynamic regulation of leukocyte beta adrenergic receptor-agonist interactions by physiological changes in circulating catecholamines. J Clin Investigat. (1983) 72:164–70. doi: 10.1172/JCI110954 PMC1129171 6308044

[38] Strange PG. Dopamine receptors: structure and function. Prog Brain Res. (1993) 99:167–79. doi: 10.1016/S0079-6123(08)61345-X 8108547

[39] Li M Zhou L Sun X Yang Y Zhang C Wang T. Dopamine, a co-regulatory component, bridges the central nervous system and the immune system. Biomed Pharmacother. (2022) 145:112458. doi: 10.1016/j.biopha.2021.112458 34847478

[40] Yaman B. Perspective chapter: the role of dopamine receptors in neuropsychiatric diseases. In: Chandra Yenisetti SC Koza Z Kumar D Singh SK Ganeshpurkar A Modi P Das A, editors. Parkinson’s Disease - Animal Models, Current Therapies and Clinical Trials. IntechOpen, Rijeka (2023).

[41] Lee TK Yankee EL. A review on Parkinson’s disease treatment. Neurosciences. (2021) 8:222. doi: 10.20517/2347-8659.2020.58

[42] Sharma D Farrar JD. Adrenergic regulation of immune cell function and inflammation. Semin Immunopathol. (2020) 42(6):709–17. doi: 10.1007/s00281-020-00829-6 PMC7678770 33219396

[43] Chavan SS Pavlov VA Tracey KJ. Mechanisms and therapeutic relevance of neuro-immune communication. Immunity. (2017) 46:927–42. doi: 10.1016/j.immuni.2017.06.008 PMC5578398 28636960

[44] Elenkov I. “Neuroendocrine effects on immune system”. In: Feingold KR Anawalt B Boyce A Chrousos G Dungan K Grossman A, editors. Endotext [Internet]. South Dartmouth (MA): MDText.com, Inc. (2000).

[45] Elenkov IJ Wilder RL Chrousos GP Vizi ES. The sympathetic nerve–an integrative interface between two supersystems: the brain and the immune system. Pharmacol Rev. (2000) 52:595–638. doi: 10.1016/S0031-6997(24)01470-4 11121511

[46] Sternberg EM. Neural regulation of innate immunity: a coordinated nonspecific host response to pathogens. Nat Rev Immunol. (2006) 6:318–28. doi: 10.1038/nri1810 PMC1783839 16557263

[47] Pavlov VA Tracey KJ. Neural regulators of innate immune responses and inflammation. Cell Mol Life Sci. (2004) 61:2322–31. doi: 10.1007/s00018-004-4102-3 PMC11138906 15378203

[48] Marino F Cosentino M. Adrenergic modulation of immune cells: an update. Amino Acids. (2013) 45:55–71. doi: 10.1007/s00726-011-1186-6 22160285

[49] Galvez I Martin-Cordero L HinChado MD Alvarez-Barrientos A Ortega E. Anti-inflammatory effect of beta2 adrenergic stimulation on circulating monocytes with a pro-inflammatory state in high-fat diet-induced obesity. Brain Behav Immun. (2019) 80:564–72. doi: 10.1016/j.bbi.2019.04.042 31055173

[50] Miksa M Das P Zhou M Wu R Dong W Ji Y. Pivotal role of the alpha(2A)-adrenoceptor in producing inflammation and organ injury in a rat model of sepsis. PloS One. (2009) 4(5):e5504. doi: 10.1371/journal.pone.0005504 19430535 PMC2677660

[51] Grisanti LA Woster AP Dahlman J Sauter ER Combs CK Porter JE. alpha1-adrenergic receptors positively regulate Toll-like receptor cytokine production from human monocytes and macrophages. J Pharmacol Exp Ther. (2011) 338:648–57. doi: 10.1124/jpet.110.178012 PMC3141906 21571945

[52] Guryanova S Ferberg A Sigmatulin I. Inflammatory response modulation by epinephrine and norepinephrine. RUDN J Med. (2023) 27:329–41. doi: 10.22363/2313-0245-2023-27-3-329-341

[53] Mossner R Lesch KP. Role of serotonin in the immune system and in neuroimmune interactions. Brain Behav Immun. (1998) 12:249–71. doi: 10.1006/brbi.1998.0532 10080856

[54] Duerschmied D Canault M Lievens D Brill A Cifuni SM Bader M. Serotonin stimulates platelet receptor shedding by tumor necrosis factor-alpha-converting enzyme (ADAM17). J Thromb Haemost. (2009) 7:1163–71. doi: 10.1111/j.1538-7836.2009.03476.x PMC3244876 19426283

[55] Fazzino F Montes C Urbina M Carreira I Lima L. Serotonin transporter is differentially localized in subpopulations of lymphocytes of major depression patients. Effect of fluoxetine on proliferation. J Neuroimmunol. (2008) 196:173–80. doi: 10.1016/j.jneuroim.2008.03.012 18462811

[56] Edgar VA Genaro AM Cremaschi G Sterin-Borda L. Fluoxetine action on murine T-lymphocyte proliferation: participation of PKC activation and calcium mobilisation. Cell Signal. (1998) 10:721–6. doi: 10.1016/S0898-6568(98)00016-3 9884023

[57] Fazzino F Urbina M Cedeno N Lima L. Fluoxetine treatment to rats modifies serotonin transporter and cAMP in lymphocytes, CD4+ and CD8+ subpopulations and interleukins 2 and 4. Int Immunopharmacol. (2009) 9:463–7. doi: 10.1016/j.intimp.2009.01.011 19189865

[58] Sacramento PM Monteiro C Dias ASO Kasahara TM Ferreira TB Hygino J. Serotonin decreases the production of Th1/Th17 cytokines and elevates the frequency of regulatory CD4(+) T-cell subsets in multiple sclerosis patients. Eur J Immunol. (2018) 48:1376–88. doi: 10.1002/eji.201847525 29719048

[59] Kanova M Kohout P. Serotonin—Its synthesis and roles in the healthy and the critically ill. Int J Mol Sci. (2021) 22:4837. doi: 10.3390/ijms22094837 34063611 PMC8124334

[60] Imamdin A van der Vorst EPC. Exploring the role of serotonin as an immune modulatory component in cardiovascular diseases. Int J Mol Sci. (2023) 24:1549. doi: 10.3390/ijms24021549 36675065 PMC9861641

[61] Wan M Ding L Wang D Han J Gao P. Serotonin: A potent immune cell modulator in autoimmune diseases. Front Immunol. (2020) 11:186. doi: 10.3389/fimmu.2020.00186 32117308 PMC7026253

[62] Freire-Garabal M Nunez MJ Balboa J Lopez-Delgado P Gallego R Garcia-Caballero T. Serotonin upregulates the activity of phagocytosis through 5-HT1A receptors. Br J Pharmacol. (2003) 139(2):457–63. doi: 10.1038/sj.bjp.0705188 PMC1573834 12770951

[63] de Las Casas-Engel M Corbi AL. Serotonin modulation of macrophage polarization: inflammation and beyond. Adv Exp Med Biol. (2014) 824:89–115. doi: 10.1007/978-3-319-07320-0_9 25038996

[64] Idzko M Panther E Stratz C Muller T Bayer H Zissel G. The serotoninergic receptors of human dendritic cells: identification and coupling to cytokine release. J Immunol. (2004) 172:6011–9. doi: 10.4049/jimmunol.172.10.6011 15128784

[65] Kim JJ Bridle BW Ghia JE Wang H Syed SN Manocha MM. Targeted inhibition of serotonin type 7 (5-HT7) receptor function modulates immune responses and reduces the severity of intestinal inflammation. J Immunol. (2013) 190:4795–804. doi: 10.4049/jimmunol.1201887 23554310

[66] Holst K Guseva D Schindler S Sixt M Braun A Chopra H. The serotonin receptor 5-HT(7)R regulates the morphology and migratory properties of dendritic cells. J Cell Sci. (2015) 128(15):2866–80. doi: 10.1242/jcs.167999 26092936

[67] Szabo A Gogolak P Koncz G Foldvari Z Pazmandi K Miltner N. Immunomodulatory capacity of the serotonin receptor 5-HT2B in a subset of human dendritic cells. Sci Rep. (2018) 8:1765. doi: 10.1038/s41598-018-20173-y 29379077 PMC5788853

[68] Chen B-R Wu T Chen T-H Wang Y. Neuroimmune interactions and their roles in neurodegenerative diseases. Fundam Res. (2024) 4:251–61. doi: 10.1016/j.fmre.2023.04.002 PMC11197660 38933502

[69] Moore SC Vaz de Castro PAS Yaqub D Jose PA Armando I. Anti-inflammatory effects of peripheral dopamine. Int J Mol Sci. (2023) 24:13816. doi: 10.3390/ijms241813816 37762126 PMC10530375

[70] Shao W Zhang S-z Tang M Zhang X-h Zhou Z Yin Y-q. Suppression of neuroinflammation by astrocytic dopamine D2 receptors via αB-crystallin. Nature. (2013) 494:90–4. doi: 10.1038/nature11748 23242137

[71] Marino F Pinoli M Rasini E Martini S Luini A Pulze L. Dopaminergic inhibition of human neutrophils is exerted through D1-like receptors and affected by bacterial infection. Immunology. (2022) 167:508–27. doi: 10.1111/imm.v167.4 35897164

[72] Levite M Chowers Y Ganor Y Besser M Hershkovits R Cahalon L. Dopamine interacts directly with its D3 and D2 receptors on normal human T cells, and activates beta1 integrin function. Eur J Immunol. (2001) 31:3504–12. doi: 10.1002/1521-4141(200112)31:12<3504::AID-IMMU3504>3.0.CO;2-F 11745370

[73] Mori M Oda T Nishiyama K Serikawa T Yamada J Ichiyama A. A single serine:pyruvate aminotransferase gene on rat chromosome 9q34-q36. Genomics. (1992) 13:686–9. doi: 10.1016/0888-7543(92)90142-F 1639396

[74] Besser MJ Ganor Y Levite M. Dopamine by itself activates either D2, D3 or D1/D5 dopaminergic receptors in normal human T-cells and triggers the selective secretion of either IL-10, TNFalpha or both. J Neuroimmunol. (2005) 169:161–71. doi: 10.1016/j.jneuroim.2005.07.013 16150496

[75] Saha B Mondal AC Basu S Dasgupta PS. Circulating dopamine level, in lung carcinoma patients, inhibits proliferation and cytotoxicity of CD4+ and CD8+ T cells by D1 dopamine receptors: an *in vitro* analysis. Int Immunopharmacol. (2001) 1:1363–74. doi: 10.1016/S1567-5769(01)00068-6 11460316

[76] Ghosh MC Mondal AC Basu S Banerjee S Majumder J Bhattacharya D. Dopamine inhibits cytokine release and expression of tyrosine kinases, Lck and Fyn in activated T cells. Int Immunopharmacol. (2003) 3:1019–26. doi: 10.1016/S1567-5769(03)00100-0 12810359

[77] Xia Q-P Cheng Z-Y He L. The modulatory role of dopamine receptors in brain neuroinflammation. Int Immunopharmacol. (2019) 76:105908. doi: 10.1016/j.intimp.2019.105908 31622861

[78] Han X Li B Ye X Mulatibieke T Wu J Dai J. Dopamine D2 receptor signalling controls inflammation in acute pancreatitis via a PP2A-dependent Akt/NF-κB signalling pathway. Br J Pharmacol. (2017) 174:4751–70. doi: 10.1111/bph.v174.24 PMC5727253 28963856

[79] Thomas Broome S Louangaphay K Keay KA Leggio GM Musumeci G Castorina A. Dopamine: an immune transmitter. Neural Regen Res. (2020) 15:2173–85. doi: 10.4103/1673-5374.284976 PMC7749467 32594028

[80] Channer B Matt SM Nickoloff-Bybel EA Pappa V Agarwal Y Wickman J. Dopamine, immunity, and disease. Pharmacol Rev. (2023) 75:62–158. doi: 10.1124/pharmrev.122.000618 36757901 PMC9832385

[81] Lee J-S Lee S-B Kim D-W Shin N Jeong S-J Yang C-H. Social isolation–related depression accelerates ethanol intake via microglia-derived neuroinflammation. Sci Adv. (2021) 7:eabj3400. doi: 10.1126/sciadv.abj3400 34739315 PMC8570606

[82] Leite JA Orellana AMM Kinoshita PF de Mello NP Scavone C Kawamoto EM. “Mechanisms of Neuroinflammation”. In: Neuroinflammation and Neurotransmission Mechanisms Involved in Neuropsychiatric Disorders. IntechOpen: London, UK: (2017). https://scholar.google.com/scholar_lookup?title=Mechanisms%20of%20Neuroinflammation&author=J.A.%20Leite&author=A.M.M.%20Orellana&author=P.F.%20Kinoshita&author=N.P.%20de%20Mello&author=C.%20Scavone&publication_year=2017&.

[83] Gao C Jiang J Tan Y Chen S. Microglia in neurodegenerative diseases: mechanism and potential therapeutic targets. Signal Transduct Target Ther. (2023) 8:359. doi: 10.1038/s41392-023-01588-0 37735487 PMC10514343

[84] Brisch R Wojtylak S Saniotis A Steiner J Gos T Kumaratilake J. The role of microglia in neuropsychiatric disorders and suicide. Eur Arch Psychiatry Clin Neurosci. (2022) 272:929–45. doi: 10.1007/s00406-021-01334-z PMC9388452 34595576

[85] Hertz L Lovatt D Goldman SA Nedergaard M. Adrenoceptors in brain: cellular gene expression and effects on astrocytic metabolism and [Ca(2+)]i. Neurochem Int. (2010) 57:411–20. doi: 10.1016/j.neuint.2010.03.019 PMC2934885 20380860

[86] Mantyh PW Rogers SD Allen CJ Catton MD Ghilardi JR Levin LA. Beta 2-adrenergic receptors are expressed by glia *in vivo* in the normal and injured central nervous system in the rat, rabbit, and human. J Neurosci. (1995) 15::152–64. doi: 10.1523/JNEUROSCI.15-01-00152.1995 PMC6578266 7823126

[87] Heneka MT Galea E Gavriluyk V Dumitrescu-Ozimek L Daeschner J O’Banion MK. Noradrenergic depletion potentiates beta -amyloid-induced cortical inflammation: implications for Alzheimer’s disease. J Neurosci. (2002) 22:2434–42. doi: 10.1523/JNEUROSCI.22-07-02434.2002 PMC6758307 11923407

[88] Bharani KL Derex R Granholm AC Ledreux A. A noradrenergic lesion aggravates the effects of systemic inflammation on the hippocampus of aged rats. PloS One. (2017) 12:e0189821. doi: 10.1371/journal.pone.0189821 29261743 PMC5736222

[89] Pugh PL Vidgeon-Hart MP Ashmeade T Culbert AA Seymour Z Perren MJ. Repeated administration of the noradrenergic neurotoxin N-(2-chloroethyl)-N-ethyl-2-bromobenzylamine (DSP-4) modulates neuroinflammation and amyloid plaque load in mice bearing amyloid precursor protein and presenilin-1 mutant transgenes. J Neuroinflamm. (2007) 4:8. doi: 10.1186/1742-2094-4-8 PMC1810243 17324270

[90] Song S Jiang L Oyarzabal EA Wilson B Li Z Shih YI. Loss of brain norepinephrine elicits neuroinflammation-mediated oxidative injury and selective caudo-rostral neurodegeneration. Mol Neurobiol. (2019) 56:2653–69. doi: 10.1007/s12035-018-1235-1 PMC6348128 30051353

[91] O’Sullivan JB Ryan KM Curtin NM Harkin A Connor TJ. Noradrenaline reuptake inhibitors limit neuroinflammation in rat cortex following a systemic inflammatory challenge: implications for depression and neurodegeneration. Int J Neuropsychopharmacol. (2009) 12:687–99. doi: 10.1017/S146114570800967X 19046481

[92] O’Sullivan JB Ryan KM Harkin A Connor TJ. Noradrenaline reuptake inhibitors inhibit expression of chemokines IP-10 and RANTES and cell adhesion molecules VCAM-1 and ICAM-1 in the CNS following a systemic inflammatory challenge. J Neuroimmunol. (2010) 220:34–42. doi: 10.1016/j.jneuroim.2009.12.007 20061033

[93] McNamee EN Ryan KM Griffin EW Gonzalez-Reyes RE Ryan KJ Harkin A. Noradrenaline acting at central beta-adrenoceptors induces interleukin-10 and suppressor of cytokine signaling-3 expression in rat brain: implications for neurodegeneration. Brain Behav Immun. (2010) 24:660–71. doi: 10.1016/j.bbi.2010.02.005 20193756

[94] Mori K Ozaki E Zhang B Yang L Yokoyama A Takeda I. Effects of norepinephrine on rat cultured microglial cells that express alpha1, alpha2, beta1 and beta2 adrenergic receptors. Neuropharmacology. (2002) 43:1026–34. doi: 10.1016/S0028-3908(02)00211-3 12423672

[95] Chang JY Liu LZ. Catecholamines inhibit microglial nitric oxide production. Brain Res Bull. (2000) 52:525–30. doi: 10.1016/S0361-9230(00)00291-4 10974492

[96] Madrigal JL Feinstein DL Dello Russo C. Norepinephrine protects cortical neurons against microglial-induced cell death. J Neurosci Res. (2005) 81:390–6. doi: 10.1002/jnr.20481 15948176

[97] Ardestani PM Evans AK Yi B Nguyen T Coutellier L Shamloo M. Modulation of neuroinflammation and pathology in the 5XFAD mouse model of Alzheimer’s disease using a biased and selective beta-1 adrenergic receptor partial agonist. Neuropharmacology. (2017) 116:371–86. doi: 10.1016/j.neuropharm.2017.01.010 PMC5385159 28089846

[98] McNamee EN Griffin EW Ryan KM Ryan KJ Heffernan S Harkin A. Noradrenaline acting at beta-adrenoceptors induces expression of IL-1beta and its negative regulators IL-1ra and IL-1RII, and drives an overall anti-inflammatory phenotype in rat cortex. Neuropharmacology. (2010) 59:37–48. doi: 10.1016/j.neuropharm.2010.03.014 20361987

[99] Frohman EM Vayuvegula B Gupta S van den Noort S. Norepinephrine inhibits gamma-interferon-induced major histocompatibility class II (Ia) antigen expression on cultured astrocytes via beta-2-adrenergic signal transduction mechanisms. Proc Natl Acad Sci U S A. (1988) 85:1292–6. doi: 10.1073/pnas.85.4.1292 PMC279753 2829222

[100] Loughlin AJ Woodroofe MN Cuzner ML. Modulation of interferon-gamma-induced major histocompatibility complex class II and Fc receptor expression on isolated microglia by transforming growth factor-beta 1, interleukin-4, noradrenaline and glucocorticoids. Immunology. (1993) 79(1):125–30. PMC1422051 8509133

[101] McNamee EN Ryan KM Kilroy D Connor TJ. Noradrenaline induces IL-1ra and IL-1 type II receptor expression in primary glial cells and protects against IL-1beta-induced neurotoxicity. Eur J Pharmacol. (2010) 626:219–28. doi: 10.1016/j.ejphar.2009.09.054 19818755

[102] Ryan KJ Griffin E Yssel JD Ryan KM McNamee EN Harkin A. Stimulation of central beta2-adrenoceptors suppresses NFkappaB activity in rat brain: a role for IkappaB. Neurochem Int. (2013) 63:368–78. doi: 10.1016/j.neuint.2013.07.006 23896303

[103] Macchi F Homberg JR Calabrese F Zecchillo C Racagni G Riva MA. Altered inflammatory responsiveness in serotonin transporter mutant rats. J Neuroinflamm. (2013) 10:116. doi: 10.1186/1742-2094-10-116 PMC3848577 24050835

[104] Miyazaki I Asanuma M. Serotonin 1A receptors on astrocytes as a potential target for the treatment of parkinson’s disease. Curr Med Chem. (2016) 23:686–700. doi: 10.2174/0929867323666160122115057 26795196 PMC4997990

[105] Guseva D Holst K Kaune B Meier M Keubler L Glage S. Serotonin 5-HT7 receptor is critically involved in acute and chronic inflammation of the gastrointestinal tract. Inflamm Bowel Dis. (2014) 20:1516–29. doi: 10.1097/MIB.0000000000000150 25072499

[106] Xing C Chen H Bi W Lei T Hang Z Du H. Targeting 5-HT is a potential therapeutic strategy for neurodegenerative diseases. Int J Mol Sci. (2024) 25:13446. doi: 10.3390/ijms252413446 39769209 PMC11679250

[107] Quintero-Villegas A Valdés-Ferrer SI. Central nervous system effects of 5-HT7 receptors: a potential target for neurodegenerative diseases. Mol Med. (2022) 28:70. doi: 10.1186/s10020-022-00497-2 35725396 PMC9208181

[108] Turkin A TuChina O Klempin F. Microglia function on precursor cells in the adult hippocampus and their responsiveness to serotonin signaling. Front Cell Dev Biol. (2021) 9:665739. doi: 10.3389/fcell.2021.665739 34109176 PMC8182052

[109] Nakagawa Y Chiba K. Role of microglial M1/M2 polarization in relapse and remission of psychiatric disorders and diseases. Pharmaceuticals. (2014) 7:1028–48. doi: 10.3390/ph7121028 PMC4276905 25429645

[110] Nagy EE Frigy A Szász JA Horváth E. Neuroinflammation and microglia/macrophage phenotype modulate the molecular background of post-stroke depression: A literature review. Exp Ther Med. (2020) 20:2510–23. doi: 10.3892/etm.2020.8933 PMC7401670 32765743

[111] Tynan RJ Weidenhofer J Hinwood M Cairns MJ Day TA Walker FR. A comparative examination of the anti-inflammatory effects of SSRI and SNRI antidepressants on LPS stimulated microglia. Brain Behav Immun. (2012) 26:469–79. doi: 10.1016/j.bbi.2011.12.011 22251606

[112] Horikawa H Kato TA Mizoguchi Y Monji A Seki Y Ohkuri T. Inhibitory effects of SSRIs on IFN-gamma induced microglial activation through the regulation of intracellular calcium. Prog Neuropsychopharmacol Biol Psychiatry. (2010) 34:1306–16. doi: 10.1016/j.pnpbp.2010.07.015 20654672

[113] Krabbe G Matyash V Pannasch U Mamer L Boddeke HW Kettenmann H. Activation of serotonin receptors promotes microglial injury-induced motility but attenuates phagocytic activity. Brain Behav Immun. (2012) 26:419–28. doi: 10.1016/j.bbi.2011.12.002 22198120

[114] Carson MJ Thomas EA Danielson PE Sutcliffe JG. The 5HT5A serotonin receptor is expressed predominantly by astrocytes in which it inhibits cAMP accumulation: a mechanism for neuronal suppression of reactive astrocytes. Glia. (1996) 17:317–26. doi: 10.1002/(SICI)1098-1136(199608)17:4<317::AID-GLIA6>3.0.CO;2-W 8856328

[115] Pousset F Fournier J Legoux P Keane P Shire D Soubrie P. Effect of serotonin on cytokine mRNA expression in rat hippocampal astrocytes. Brain Res Mol Brain Res. (1996) 38:54–62. doi: 10.1016/0169-328X(95)00324-L 8737667

[116] Liu RP Zou M Wang JY Zhu JJ Lai JM Zhou LL. Paroxetine ameliorates lipopolysaccharide-induced microglia activation via differential regulation of MAPK signaling. J Neuroinflamm. (2014) 11:47. doi: 10.1186/1742-2094-11-47 PMC3995780 24618100

[117] Lim CM Kim SW Park JY Kim C Yoon SH Lee JK. Fluoxetine affords robust neuroprotection in the postischemic brain via its anti-inflammatory effect. J Neurosci Res. (2009) 87:1037–45. doi: 10.1002/jnr.21899 18855941

[118] Chung YC Kim SR Jin BK. Paroxetine prevents loss of nigrostriatal dopaminergic neurons by inhibiting brain inflammation and oxidative stress in an experimental model of Parkinson’s disease. J Immunol. (2010) 185:1230–7. doi: 10.4049/jimmunol.1000208 20566832

[119] Leggio GM Salomone S Bucolo C Platania C Micale V Caraci F. Dopamine D(3) receptor as a new pharmacological target for the treatment of depression. Eur J Pharmacol. (2013) 719:25–33. doi: 10.1016/j.ejphar.2013.07.022 23872400

[120] Mishra A Singh S Shukla S. Physiological and functional basis of dopamine receptors and their role in neurogenesis: possible implication for parkinson’s disease. J Exp Neurosci. (2018) 12:1179069518779829. doi: 10.1177/1179069518779829 29899667 PMC5985548

[121] Kuric E Wieloch T Ruscher K. Dopamine receptor activation increases glial cell line-derived neurotrophic factor in experimental stroke. Exp Neurol. (2013) 247:202–8. doi: 10.1016/j.expneurol.2013.04.016 23664961

[122] Huck JH Freyer D Bottcher C Mladinov M Muselmann-Genschow C Thielke M. *De novo* expression of dopamine D2 receptors on microglia after stroke. J Cereb Blood Flow Metab. (2015) 35:1804–11. doi: 10.1038/jcbfm.2015.128 PMC4635235 26104289

[123] Feng Y Lu Y. Immunomodulatory effects of dopamine in inflammatory diseases. Front Immunol. (2021) 12:663102. doi: 10.3389/fimmu.2021.663102 33897712 PMC8063048

[124] Zhang Y Chen Y Wu J Manaenko A Yang P Tang J. Activation of dopamine D2 receptor suppresses neuroinflammation through αB-crystalline by inhibition of NF-κB nuclear translocation in experimental ICH mice model. Stroke. (2015) 46:2637–46. doi: 10.1161/STROKEAHA.115.009792 PMC4550518 26251254

[125] Yan Y Jiang W Liu L Wang X Ding C Tian Z. Dopamine controls systemic inflammation through inhibition of NLRP3 inflammasome. Cell. (2015) 160:62–73. doi: 10.1016/j.cell.2014.11.047 25594175

[126] Schroder K Tschopp J. The inflammasomes. Cell. (2010) 140:821–32. doi: 10.1016/j.cell.2010.01.040 20303873

[127] Wang T Nowrangi D Yu L Lu T Tang J Han B. Activation of dopamine D1 receptor decreased NLRP3-mediated inflammation in intracerebral hemorrhage mice. J Neuroinflamm. (2018) 15:2. doi: 10.1186/s12974-017-1039-7 PMC5753458 29301581

[128] Wang B Chen T Xue L Wang J Jia Y Li G. Methamphetamine exacerbates neuroinflammatory response to lipopolysaccharide by activating dopamine D1-like receptors. Int Immunopharmacol. (2019) 73:1–9. doi: 10.1016/j.intimp.2019.04.053 31078920

[129] Malmberg A Jackson DM Eriksson A Mohell N. Unique binding characteristics of antipsychotic agents interacting with human dopamine D2A, D2B, and D3 receptors. Mol Pharmacol. (1993) 43:749–54. doi: 10.1016/S0026-895X(25)13652-3 8099194

[130] Strange PG. Antipsychotic drugs: importance of dopamine receptors for mechanisms of therapeutic actions and side effects. Pharmacol Rev. (2001) 53:119–33. doi: 10.1016/S0031-6997(24)01483-2 11171942

[131] Sunahara RK Guan HC O’Dowd BF Seeman P Laurier LG Ng G. Cloning of the gene for a human dopamine D5 receptor with higher affinity for dopamine than D1. Nature. (1991) 350:614–9. doi: 10.1038/350614a0 1826762

[132] Wu WL Burnett DA Spring R Greenlee WJ Smith M Favreau L. Dopamine D1/D5 receptor antagonists with improved pharmacokinetics: design, synthesis, and biological evaluation of phenol bioisosteric analogues of benzazepine D1/D5 antagonists. J Med Chem. (2005) 48:680–93. doi: 10.1021/jm030614p 15689153

[133] Pacheco R. Targeting dopamine receptor D3 signalling in inflammation. Oncotarget. (2017) 8:7224–5. doi: 10.18632/oncotarget.14601 PMC5352314 28086229

[134] Gonzalez H Contreras F Prado C Elgueta D Franz D Bernales S. Dopamine receptor D3 expressed on CD4+ T cells favors neurodegeneration of dopaminergic neurons during Parkinson’s disease. J Immunol. (2013) 190(10):5048–56. doi: 10.4049/jimmunol.1203121 23589621

[135] Moraga-Amaro R Gonzalez H Pacheco R Stehberg J. Dopamine receptor D3 deficiency results in chronic depression and anxiety. Behav Brain Res. (2014) 274:186–93. doi: 10.1016/j.bbr.2014.07.055 25110304

[136] Wang J Lai S Li G Zhou T Wang B Cao F. Microglial activation contributes to depressive-like behavior in dopamine D3 receptor knockout mice. Brain Behav Immun. (2020) 83:226–38. doi: 10.1016/j.bbi.2019.10.016 31626970

[137] Corrigan M O'Rourke AM Moran B Fletcher JM Harkin A. Inflammation in the pathogenesis of depression: a disorder of neuroimmune origin. Neuronal Signal. (2023) 7(2):NS20220054. doi: 10.1042/NS20220054 PMC10345431 37457896

[138] Wang J Lai S Li G Zhou T Wang B Cao F. Microglial activation contributes to depressive-like behavior in dopamine D3 receptor knockout mice. Brain Behav Immun. (2020) 83:226–38. doi: 10.1016/j.bbi.2019.10.016 31626970

[139] Chan KL Cathomas F Russo SJ. Central and peripheral inflammation link metabolic syndrome and major depressive disorder. Physiol (Bethesda). (2019) 34:123–33. doi: 10.1152/physiol.00047.2018 PMC6586832 30724127

[140] Grygiel-Gorniak B Limphaibool N Puszczewicz M. Cytokine secretion and the risk of depression development in patients with connective tissue diseases. Psychiatry Clin Neurosci. (2019) 73:302–16. doi: 10.1111/pcn.2019.73.issue-6 30719813

[141] Pisanu A Boi L Mulas G Spiga S Fenu S Carta AR. Neuroinflammation in L-DOPA-induced dyskinesia: beyond the immune function. J Neural Transm (Vienna). (2018) 125:1287–97. doi: 10.1007/s00702-018-1874-4 29541852

[142] Miller AH Haroon E Raison CL Felger JC. Cytokine targets in the brain: impact on neurotransmitters and neurocircuits. Depress Anxiety. (2013) 30:297–306. doi: 10.1002/da.2013.30.issue-4 23468190 PMC4141874

[143] Dantzer R. Neuroimmune interactions: from the brain to the immune system and vice versa. Physiol Rev. (2018) 98:477–504. doi: 10.1152/physrev.00039.2016 29351513 PMC5866360

[144] Beurel E Toups M Nemeroff CB. The bidirectional relationship of depression and inflammation: double trouble. Neuron. (2020) 107:234–56. doi: 10.1016/j.neuron.2020.06.002 PMC7381373 32553197

[145] Felger JC Lotrich FE. Inflammatory cytokines in depression: neurobiological mechanisms and therapeutic implications. Neuroscience. (2013) 246:199–229. doi: 10.1016/j.neuroscience.2013.04.060 23644052 PMC3741070

[146] Anderson G Kubera M Duda W Lason W Berk M Maes M. Increased IL-6 trans-signaling in depression: focus on the tryptophan catabolite pathway, melatonin and neuroprogression. Pharmacol Rep. (2013) 65:1647–54. doi: 10.1016/S1734-1140(13)71526-3 24553013

[147] Jurgens B Hainz U Fuchs D Felzmann T Heitger A. Interferon-gamma-triggered indoleamine 2,3-dioxygenase competence in human monocyte-derived dendritic cells induces regulatory activity in allogeneic T cells. Blood. (2009) 114(15):3235–43. doi: 10.1182/blood-2008-12-195073 19625705

[148] Babcock TA Carlin JM. Transcriptional activation of indoleamine dioxygenase by interleukin 1 and tumor necrosis factor alpha in interferon-treated epithelial cells. Cytokine. (2000) 12:588–94. doi: 10.1006/cyto.1999.0661 10843733

[149] Robinson CM Shirey KA Carlin JM. Synergistic transcriptional activation of indoleamine dioxygenase by IFN-gamma and tumor necrosis factor-alpha. J Inter Cytokine Res. (2003) 23:413–21. doi: 10.1089/107999003322277829 PMC1488822 13678429

[150] O’Connor JC Andre C Wang Y Lawson MA Szegedi SS Lestage J. Interferon-gamma and tumor necrosis factor-alpha mediate the upregulation of indoleamine 2,3-dioxygenase and the induction of depressive-like behavior in mice in response to bacillus Calmette-Guerin. J Neurosci. (2009) 29:4200–9. doi: 10.1523/JNEUROSCI.5032-08 PMC2835569 19339614

[151] Guillemin GJ Smythe G Takikawa O Brew BJ. Expression of indoleamine 2,3-dioxygenase and production of quinolinic acid by human microglia, astrocytes, and neurons. Glia. (2005) 49:15–23. doi: 10.1002/glia.20090 15390107

[152] Fanet H Capuron L Castanon N Calon F Vancassel S. Tetrahydrobioterin (BH4) pathway: from metabolism to neuropsychiatry. Curr Neuropharmacol. (2021) 19(5):591–609. doi: 10.2174/1570159X18666200729103529 32744952 PMC8573752

[153] Kappock TJ Caradonna JP. Pterin-dependent amino acid hydroxylases. Chem Rev. (1996) 96:2659–756. doi: 10.1021/cr9402034 11848840

[154] Calka J. The role of nitric oxide in the hypothalamic control of LHRH and oxytocin release, sexual behavior and aging of the LHRH and oxytocin neurons. Folia Histochem Cytobiol. (2006) 44(1):3–12. 16584085

[155] Walter R Blau N Kierat L Schaffner A Schoedon G. Effects of activating and deactivating cytokines on the functionally linked tetrahydrobiopterin. No pathways in vascular smooth muscle cells. Immunol Lett. (1996) 54:25–9. doi: 10.1016/S0165-2478(96)02638-7 9030978

[156] Hattori Y Nakanishi N Kasai K Shimoda SI. GTP cyclohydrolase I mRNA induction and tetrahydrobiopterin synthesis in human endothelial cells. Biochim Biophys Acta. (1997) 1358:61–6. doi: 10.1016/S0167-4889(97)00052-9 9296522

[157] Vann LR Payne SG Edsall LC Twitty S Spiegel S Milstien S. Involvement of sphingosine kinase in TNF-alpha-stimulated tetrahydrobiopterin biosynthesis in C6 glioma cells. J Biol Chem. (2002) 277:12649–56. doi: 10.1074/jbc.M109111200 11815603

[158] Neurauter G Schrocksnadel K Scholl-Burgi S Sperner-Unterweger B Schubert C Ledochowski M. Chronic immune stimulation correlates with reduced phenylalanine turnover. Curr Drug Metab. (2008) 9:622–7. doi: 10.2174/138920008785821738 18781914

[159] Joseph A Kumar A O’Connell NA Agarwal RK Gwosdow AR. Interleukin-1 alpha stimulates dopamine release by activating type II protein kinase A in PC-12 cells. Am J Physiol. (1995) 269:E1083–8. doi: 10.1152/ajpendo.1995.269.6.E1083 8572200

[160] Shintani F Kanba S Nakaki T Nibuya M Kinoshita N Suzuki E. Interleukin-1 beta augments release of norepinephrine, dopamine, and serotonin in the rat anterior hypothalamus. J Neurosci. (1993) 13:3574–81. doi: 10.1523/JNEUROSCI.13-08-03574.1993 PMC6576546 8393485

[161] Capuron L Pagnoni G Drake DF Woolwine BJ Spivey JR Crowe RJ. Dopaminergic mechanisms of reduced basal ganglia responses to hedonic reward during interferon alfa administration. Arch Gen Psychiatry. (2012) 69:1044–53. doi: 10.1001/archgenpsychiatry.2011.2094 PMC3640298 23026954

[162] Wu HQ Rassoulpour A Schwarcz R. Kynurenic acid leads, dopamine follows: a new case of volume transmission in the brain? J Neural Transm (Vienna). (2007) 114(1):33–41. doi: 10.1007/s00702-006-0562-y 16932989

[163] Penmatsa A Wang KH Gouaux E. X-ray structure of dopamine transporter elucidates antidepressant mechanism. Nature. (2013) 503:85–90. doi: 10.1038/nature12533 24037379 PMC3904663

[164] Wang KH Penmatsa A Gouaux E. Neurotransmitter and psychostimulant recognition by the dopamine transporter. Nature. (2015) 521:322–7. doi: 10.1038/nature14431 PMC4469479 25970245

[165] Penmatsa A Wang KH Gouaux E. X-ray structures of Drosophila dopamine transporter in complex with nisoxetine and reboxetine. Nat Struct Mol Biol. (2015) 22:506–8. doi: 10.1038/nsmb.3029 PMC4608549 25961798

[166] Coleman JA Yang D Zhao Z Wen PC Yoshioka C Tajkhorshid E. Serotonin transporter-ibogaine complexes illuminate mechanisms of inhibition and transport. Nature. (2019) 569:141–5. doi: 10.1038/s41586-019-1135-1 PMC6750207 31019304

[167] Coleman JA Gouaux E. Structural basis for recognition of diverse antidepressants by the human serotonin transporter. Nat Struct Mol Biol. (2018) 25:170–5. doi: 10.1038/s41594-018-0026-8 PMC5962350 29379174

[168] Coleman JA Green EM Gouaux E. X-ray structures and mechanism of the human serotonin transporter. Nature. (2016) 532:334–9. doi: 10.1038/nature17629 PMC4898786 27049939

[169] Cheng MH Bahar I. Monoamine transporters: structure, intrinsic dynamics and allosteric regulation. Nat Struct Mol Biol. (2019) 26:545–56. doi: 10.1038/s41594-019-0253-7 PMC6712983 31270469

[170] Ramamoorthy S Ramamoorthy JD Prasad PD Bhat GK Mahesh VB Leibach FH. Regulation of the human serotonin transporter by interleukin-1 beta. Biochem Biophys Res Commun. (1995) 216:560–7. doi: 10.1006/bbrc.1995.2659 7488148

[171] Morikawa O Sakai N Obara H Saito N. Effects of interferon-alpha, interferon-gamma and cAMP on the transcriptional regulation of the serotonin transporter. Eur J Pharmacol. (1998) 349:317–24. doi: 10.1016/S0014-2999(98)00187-3 9671113

[172] Mossner R Heils A Stober G Okladnova O Daniel S Lesch KP. Enhancement of serotonin transporter function by tumor necrosis factor alpha but not by interleukin-6. Neurochem Int. (1998) 33:251–4. doi: 10.1016/S0197-0186(98)00026-6 9759920

[173] Mossner R Daniel S Schmitt A Albert D Lesch KP. Modulation of serotonin transporter function by interleukin-4. Life Sci. (2001) 68:873–80. doi: 10.1016/S0024-3205(00)00992-9 11213357

[174] Wu Y Na X Zang Y Cui Y Xin W Pang R. Upregulation of tumor necrosis factor-alpha in nucleus accumbens attenuates morphine-induced rewarding in a neuropathic pain model. Biochem Biophys Res Commun. (2014) 449:502–7. doi: 10.1016/j.bbrc.2014.05.025 24845379

[175] Bermingham DP Blakely RD. Kinase-dependent regulation of monoamine neurotransmitter transporters. Pharmacol Rev. (2016) 68:888–953. doi: 10.1124/pr.115.012260 27591044 PMC5050440

[176] Miller AH Maletic V Raison CL. Inflammation and its discontents: the role of cytokines in the pathophysiology of major depression. Biol Psychiatry. (2009) 65:732–41. doi: 10.1016/j.biopsych.2008.11.029 PMC2680424 19150053

[177] Zhu CB Blakely RD Hewlett WA. The proinflammatory cytokines interleukin-1beta and tumor necrosis factor-alpha activate serotonin transporters. Neuropsychopharmacology. (2006) 31:2121–31. doi: 10.1038/sj.npp.1301029 16452991

[178] Terao A Oikawa M Saito M. Cytokine-induced change in hypothalamic norepinephrine turnover: involvement of corticotropin-releasing hormone and prostaglandins. Brain Res. (1993) 622:257–61. doi: 10.1016/0006-8993(93)90826-9 8242363

[179] Hayley S Brebner K Lacosta S Merali Z Anisman H. Sensitization to the effects of tumor necrosis factor-alpha: neuroendocrine, central monoamine, and behavioral variations. J Neurosci. (1999) 19:5654–65. doi: 10.1523/JNEUROSCI.19-13-05654.1999 PMC6782322 10377371

[180] De La Garza R 2nd Asnis GM. The non-steroidal anti-inflammatory drug diclofenac sodium attenuates IFN-alpha induced alterations to monoamine turnover in prefrontal cortex and hippocampus. Brain Res. (2003) 977:70–9. doi: 10.1016/S0006-8993(03)02757-4 12788515

[181] Litteljohn D Rudyk C Dwyer Z Farmer K Fortin T Hayley S. The impact of murine LRRK2 G2019S transgene overexpression on acute responses to inflammatory challenge. Brain Behav Immun. (2018) 67:246–56. doi: 10.1016/j.bbi.2017.09.002 28893563

[182] Adzic M Brkic Z Mitic M Francija E Jovicic MJ Radulovic J. Therapeutic strategies for treatment of inflammation-related depression. Curr Neuropharmacol. (2018) 16:176–209. doi: 10.2174/1570159X15666170828163048 28847294 PMC5883379

[183] Haroon E Raison CL Miller AH. Psychoneuroimmunology meets neuropsychopharmacology: translational implications of the impact of inflammation on behavior. Neuropsychopharmacology. (2012) 37:137–62. doi: 10.1038/npp.2011.205 PMC3238082 21918508

[184] Bhattacharya A Derecki NC Lovenberg TW Drevets WC. Role of neuro-immunological factors in the pathophysiology of mood disorders. Psychopharmacol (Berl). (2016) 233:1623–36. doi: 10.1007/s00213-016-4214-0 26803500

[185] Krishnadas R Cavanagh J. Depression: an inflammatory illness? J Neurol Neurosurg Psychiatry. (2012) 83(5):495–502. doi: 10.1136/jnnp-2011-301779 22423117

[186] Bhattacharya A Derecki NC Lovenberg TW Drevets WC. Role of neuro-immunological factors in the pathophysiology of mood disorders. Psychopharmacology (Berl). (2016) 233(9):1623–36. doi: 10.1007/s00213-016-4214-0 26803500

[187] Hassamal S. Chronic stress, neuroinflammation, and depression: an overview of pathophysiological mechanisms and emerging anti-inflammatories. Front Psychiatry. (2023) 14:1130989. doi: 10.3389/fpsyt.2023.1130989 37252156 PMC10213648

[188] Roman M Irwin MR. Novel neuroimmunologic therapeutics in depression: A clinical perspective on what we know so far. Brain Behav Immun. (2020) 83:7–21. doi: 10.1016/j.bbi.2019.09.016 PMC6940145 31550500

[189] Puentes-Orozco M Albarracin SL Velásquez MM. Neuroinflammation and major depressive disorder: astrocytes at the crossroads. Front Cell Neurosci. (2024) 18:1504555. doi: 10.3389/fncel.2024.1504555 39650796 PMC11620873

[190] Zhang MM Guo MX Zhang QP Chen XQ Li NZ Liu Q. IL-1R/C3aR signaling regulates synaptic pruning in the prefrontal cortex of depression. Cell Biosci. (2022) 12:90. doi: 10.1186/s13578-022-00832-4 35715851 PMC9205119

[191] Gutierrez EG Banks WA Kastin AJ. Murine tumor necrosis factor alpha is transported from blood to brain in the mouse. J Neuroimmunol. (1993) 47:169–76. doi: 10.1016/0165-5728(93)90027-V 8370768

[192] Gasquoine PG. Contributions of the insula to cognition and emotion. Neuropsychol Rev. (2014) 24:77–87. doi: 10.1007/s11065-014-9246-9 24442602

[193] Chen P Chen F Chen G Zhong S Gong J Zhong H. Inflammation is associated with decreased functional connectivity of insula in unmedicated bipolar disorder. Brain Behav Immun. (2020) 89:615–22. doi: 10.1016/j.bbi.2020.07.004 32688026

[194] Numakawa T Richards M Nakajima S Adachi N Furuta M Odaka H. The role of brain-derived neurotrophic factor in comorbid depression: possible linkage with steroid hormones, cytokines, and nutrition. Front Psychiatry. (2014) 5:136. doi: 10.3389/fpsyt.2014.00136 25309465 PMC4175905

[195] Zunszain PA Anacker C Cattaneo A Carvalho LA Pariante CM. Glucocorticoids, cytokines and brain abnormalities in depression. Prog Neuropsychopharmacol Biol Psychiatry. (2011) 35:722–9. doi: 10.1016/j.pnpbp.2010.04.011 PMC3513408 20406665

[196] Bergamini G Mechtersheimer J Azzinnari D Sigrist H Buerge M Dallmann R. Chronic social stress induces peripheral and central immune activation, blunted mesolimbic dopamine function, and reduced reward-directed behaviour in mice. Neurobiol Stress. (2018) 8:42–56. doi: 10.1016/j.ynstr.2018.01.004 29888303 PMC5991330

[197] Bekhbat M Li Z Mehta ND Treadway MT Lucido MJ Woolwine BJ. Functional connectivity in reward circuitry and symptoms of anhedonia as therapeutic targets in depression with high inflammation: evidence from a dopamine challenge study. Mol Psychiatry. (2022) 27:4113–21. doi: 10.1038/s41380-022-01715-3 PMC9718669 35927580

[198] Belujon P Grace AA. Dopamine system dysregulation in major depressive disorders. Int J Neuropsychopharmacol. (2017) 20:1036–46. doi: 10.1093/ijnp/pyx056 PMC5716179 29106542

[199] Robson MJ Quinlan MA Blakely RD. Immune system activation and depression: roles of serotonin in the central nervous system and periphery. ACS Chem Neurosci. (2017) 8:932–42. doi: 10.1021/acschemneuro.6b00412 28345868

[200] Jha MK Minhajuddin A Gadad BS Greer TL Mayes TL Trivedi MH. Interleukin 17 selectively predicts better outcomes with bupropion-SSRI combination: Novel T cell biomarker for antidepressant medication selection. Brain Behav Immun. (2017) 66:103–10. doi: 10.1016/j.bbi.2017.07.005 PMC5699207 28698115

[201] Lucido MJ Dunlop BW. Emerging medications for treatment-resistant depression: A review with perspective on mechanisms and challenges. Brain Sci. (2025) 15(2):161. doi: 10.3390/brainsci15020161 40002494 PMC11853532

[202] Dziedzicka-Wasylewska M Willner P Papp M. Changes in dopamine receptor mRNA expression following chronic mild stress and chronic antidepressant treatment. Behav Pharmacol. (1997) 8:607–18. doi: 10.1097/00008877-199711000-00017 9832973

[203] Kram ML Kramer GL Ronan PJ Steciuk M Petty F. Dopamine receptors and learned helplessness in the rat: An autoradiographic study. Prog Neuropsychopharmacol Biol Psychiatry. (2002) 26:639–45. doi: 10.1016/S0278-5846(01)00222-6 12188094

[204] Bai S Guo W Feng Y Deng H Li G Nie H. Efficacy and safety of anti-inflammatory agents for the treatment of major depressive disorder: a systematic review and meta-analysis of randomised controlled trials. J Neurol Neurosurg Psychiatry. (2020) 91(1):21–32. doi: 10.1136/jnnp-2019-320912 31658959

[205] Brymer KJ Fenton EY Kalynchuk LE Caruncho HJ. Peripheral etanercept administration normalizes behavior, hippocampal neurogenesis, and hippocampal reelin and GABAA receptor expression in a preclinical model of depression. Front Pharmacol. (2018) 9:121. doi: 10.3389/fphar.2018.00121 29515447 PMC5826281

[206] Warner-Schmidt JL Vanover KE Chen EY Marshall JJ Greengard P. Antidepressant effects of selective serotonin reuptake inhibitors (SSRIs) are attenuated by antiinflammatory drugs in mice and humans. Proc Natl Acad Sci U S A. (2011) 108:9262–7. doi: 10.1073/pnas.1104836108 PMC3107316 21518864

[207] Martinowich K Lu B. Interaction between BDNF and serotonin: role in mood disorders. Neuropsychopharmacology. (2008) 33:73–83. doi: 10.1038/sj.npp.1301571 17882234

[208] Catena-Dell’Osso M Rotella F Dell’Osso A Fagiolini A Marazziti D. Inflammation, serotonin and major depression. Curr Drug Targets. (2013) 14:571–7. doi: 10.2174/13894501113149990154 23531160

[209] Descarries L Riad M. Effects of the antidepressant fluoxetine on the subcellular localization of 5-HT1A receptors and SERT. Philos Trans R Soc B: Biol Sci. (2012) 367:2416–25. doi: 10.1098/rstb.2011.0361 PMC3405674 22826342

[210] freeman@ roslin. ed. ac. uk MICRTGACCSMBMWPCMHNAHDABETFTCt. Effects of anti-inflammatory drugs on the expression of tryptophan-metabolism genes by human macrophages. J Leukocyte Biol. (2018) 103:681–92. doi: 10.1002/JLB.3A0617-261R PMC5918594 29377288

[211] Molero P Ramos-Quiroga J Martin-Santos R Calvo-Sánchez E Gutiérrez-Rojas L Meana J. Antidepressant efficacy and tolerability of ketamine and esketamine: a critical review. CNS Drugs. (2018) 32:411–20. doi: 10.1007/s40263-018-0519-3 29736744

[212] Richardson B MacPherson A Bambico F. Neuroinflammation and neuroprogression in depression: Effects of alternative drug treatments. Brain Behav Immun Health. (2022) 26:100554. doi: 10.1016/j.bbih.2022.100554 36388140 PMC9663329

[213] Šimić G Babić Leko M Wray S Harrington CR Delalle I Jovanov-Milošević N. Monoaminergic neuropathology in Alzheimer’s disease. Prog Neurobiol. (2017) 151:101–38. doi: 10.1016/j.pneurobio.2016.04.001 PMC5061605 27084356

[214] Matthews KL Chen CP Esiri MM Keene J Minger SL Francis PT. Noradrenergic changes, aggressive behavior, and cognition in patients with dementia. Biol Psychiatry. (2002) 51:407–16. doi: 10.1016/S0006-3223(01)01235-5 11904135

[215] Gannon M Wang Q. Complex noradrenergic dysfunction in Alzheimer’s disease: Low norepinephrine input is not always to blame. Brain Res. (2019) 1702:12–6. doi: 10.1016/j.brainres.2018.01.001 PMC6855395 29307592

[216] Szot P White SS Greenup JL Leverenz JB Peskind ER Raskind MA. Changes in adrenoreceptors in the prefrontal cortex of subjects with dementia: evidence of compensatory changes. Neuroscience. (2007) 146:471–80. doi: 10.1016/j.neuroscience.2007.01.031 PMC3399726 17324522

[217] Chen Y Peng Y Che P Gannon M Liu Y Li L. α(2A) adrenergic receptor promotes amyloidogenesis through disrupting APP-SorLA interaction. Proc Natl Acad Sci U S A. (2014) 111:17296–301. doi: 10.1073/pnas.1409513111 PMC4260556 25404298

[218] Zhang F Gannon M Chen Y Yan S Zhang S Feng W. β-amyloid redirects norepinephrine signaling to activate the pathogenic GSK3β/tau cascade. Sci Transl Med. (2020) 12(526):eaay6931. doi: 10.1126/scitranslmed.aay6931 31941827 PMC7891768

[219] Gutiérrez IL Dello Russo C Novellino F Caso JR García-Bueno B Leza JC. Noradrenaline in alzheimer’s disease: A new potential therapeutic target. Int J Mol Sci. (2022) 23(11):6143. doi: 10.3390/ijms23116143 35682822 PMC9181823

[220] Ni Y Zhao X Bao G Zou L Teng L Wang Z. Activation of beta2-adrenergic receptor stimulates gamma-secretase activity and accelerates amyloid plaque formation. Nat Med. (2006) 12:1390–6. doi: 10.1038/nm1485 17115048

[221] Heneka MT Kummer MP Latz E. Innate immune activation in neurodegenerative disease. Nat Rev Immunol. (2014) 14:463–77. doi: 10.1038/nri3705 24962261

[222] Savonije K Weaver DF. The role of tryptophan metabolism in alzheimer’s disease. Brain Sci. (2023) 13:292. doi: 10.3390/brainsci13020292 36831835 PMC9954102

[223] Capuron L Neurauter G Musselman DL Lawson DH Nemeroff CB Fuchs D. Interferon-alpha–induced changes in tryptophan metabolism: relationship to depression and paroxetine treatment. Biol Psychiatry. (2003) 54:906–14. doi: 10.1016/S0006-3223(03)00173-2 14573318

[224] Miller AH. Mechanisms of cytokine-induced behavioral changes: Psychoneuroimmunology at the translational interface. Brain Behav Immun. (2009) 23:149–58. doi: 10.1016/j.bbi.2008.08.006 PMC2745948 18793712

[225] Rhie SJ Jung EY Shim I. The role of neuroinflammation on pathogenesis of affective disorders. J Exerc Rehabil. (2020) 16:2–9. doi: 10.12965/jer.2040016.008 32161729 PMC7056473

[226] Pless A Ware D Saggu S Rehman H Morgan J Wang Q. Understanding neuropsychiatric symptoms in Alzheimer’s disease: challenges and advances in diagnosis and treatment. Front Neurosci. (2023) 17. doi: 10.3389/fnins.2023.1263771 PMC10508352 37732300

[227] Scassellati C Galoforo AC Esposito C Ciani M Ricevuti G Bonvicini C. Promising intervention approaches to potentially resolve neuroinflammation and steroid hormones alterations in alzheimer’s disease and its neuropsychiatric symptoms. Aging Dis. (2021) 12:1337–57. doi: 10.14336/AD.2021.0122 PMC8279527 34341712

[228] Hansen DV Hanson JE Sheng M. Microglia in alzheimer’s disease. J Cell Biol. (2018) 217:459–72. doi: 10.1083/jcb.201709069 PMC5800817 29196460

[229] Harry GJ Kraft AD. Neuroinflammation and microglia: considerations and approaches for neurotoxicity assessment. Expert Opin Drug Metab Toxicol. (2008) 4:1265–77. doi: 10.1517/17425255.4.10.1265 PMC2658618 18798697

[230] Hashimoto K Malchow B Falkai P Schmitt A. Glutamate modulators as potential therapeutic drugs in schizophrenia and affective disorders. Eur Arch Psychiatry Clin Neurosci. (2013) 263:367–77. doi: 10.1007/s00406-013-0399-y 23455590

[231] Metaxas A Anzalone M Vaitheeswaran R Petersen S Landau AM Finsen B. Neuroinflammation and amyloid-beta 40 are associated with reduced serotonin transporter (SERT) activity in a transgenic model of familial Alzheimer’s disease. Alzheimers Res Ther. (2019) 11:38. doi: 10.1186/s13195-019-0491-2 31043179 PMC6495598

[232] Zhang B Gaiteri C Bodea LG Wang Z McElwee J Podtelezhnikov AA. Integrated systems approach identifies genetic nodes and networks in late-onset Alzheimer’s disease. Cell. (2013) 153:707–20. doi: 10.1016/j.cell.2013.03.030 PMC3677161 23622250

[233] Kinney JW Bemiller SM Murtishaw AS Leisgang AM Salazar AM Lamb BT. Inflammation as a central mechanism in Alzheimer’s disease. Alzheimers Dement (N Y). (2018) 4:575–90. doi: 10.1016/j.trci.2018.06.014 PMC6214864 30406177

[234] Zotova E Bharambe V Cheaveau M Morgan W Holmes C Harris S. Inflammatory components in human Alzheimer’s disease and after active amyloid-β42 immunization. Brain. (2013) 136:2677–96. doi: 10.1093/brain/awt210 23943781

[235] Schaffer Aguzzoli C Ferreira PCL Povala G Ferrari-Souza JP Bellaver B Soares Katz C. Neuropsychiatric symptoms and microglial activation in patients with alzheimer disease. JAMA Netw Open. (2023) 6:e2345175–e. doi: 10.1001/jamanetworkopen.2023.45175 PMC10682836 38010651

[236] Klawonn AM Fritz M Castany S Pignatelli M Canal C Similä F. Microglial activation elicits a negative affective state through prostaglandin-mediated modulation of striatal neurons. Immunity. (2021) 54:225–34.e6. doi: 10.1016/j.immuni.2020.12.016 33476547

[237] El Khoury JB Moore KJ Means TK Leung J Terada K Toft M. CD36 mediates the innate host response to beta-amyloid. J Exp Med. (2003) 197:1657–66. doi: 10.1084/jem.20021546 PMC2193948 12796468

[238] Mawuenyega KG Sigurdson W Ovod V Munsell L Kasten T Morris JC. Decreased clearance of CNS beta-amyloid in Alzheimer’s disease. Science. (2010) 330:1774. doi: 10.1126/science.1197623 21148344 PMC3073454

[239] Streit WJ Braak H Xue QS Bechmann I. Dystrophic (senescent) rather than activated microglial cells are associated with tau pathology and likely precede neurodegeneration in Alzheimer’s disease. Acta Neuropathol. (2009) 118:475–85. doi: 10.1007/s00401-009-0556-6 PMC2737117 19513731

[240] Dhapola R Hota SS Sarma P Bhattacharyya A Medhi B Reddy DH. Recent advances in molecular pathways and therapeutic implications targeting neuroinflammation for Alzheimer’s disease. Inflammopharmacology. (2021) 29:1669–81. doi: 10.1007/s10787-021-00889-6 PMC8608577 34813026

[241] Jadhav D Saraswat N Vyawahare N Shirode D. Targeting the molecular web of Alzheimer’s disease: unveiling pathways for effective pharmacotherapy. Egyptian J Neurol Psychiatry Neurosurg. (2024) 60:7. doi: 10.1186/s41983-023-00775-8

[242] Cummings J. Anti-amyloid monoclonal antibodies are transformative treatments that redefine Alzheimer’s disease therapeutics. Drugs. (2023) 83:569–76. doi: 10.1007/s40265-023-01858-9 PMC10195708 37060386

[243] De Virgilio A Greco A Fabbrini G Inghilleri M Rizzo MI Gallo A. Parkinson’s disease: Autoimmunity and neuroinflammation. Autoimmun Rev. (2016) 15:1005–11. doi: 10.1016/j.autrev.2016.07.022 27497913

[244] Jellinger KA. Basic mechanisms of neurodegeneration: a critical update. J Cell Mol Med. (2010) 14:457–87. doi: 10.1111/j.1582-4934.2010.01010.x PMC3823450 20070435

[245] Block ML Zecca L Hong J-S. Microglia-mediated neurotoxicity: uncovering the molecular mechanisms. Nat Rev Neurosci. (2007) 8:57–69. doi: 10.1038/nrn2038 17180163

[246] Troncoso-Escudero P Parra A Nassif M Vidal RL. Outside in: unraveling the role of neuroinflammation in the progression of parkinson’s disease. Front Neurol. (2018) 9. doi: 10.3389/fneur.2018.00860 PMC6232883 30459700

[247] Sarkar C Basu B Chakroborty D Dasgupta PS Basu S. The immunoregulatory role of dopamine: an update. Brain Behav Immun. (2010) 24:525–8. doi: 10.1016/j.bbi.2009.10.015 PMC2856781 19896530

[248] Boka G Anglade P Wallach D Javoy-Agid F Agid Y Hirsch EC. Immunocytochemical analysis of tumor necrosis factor and its receptors in Parkinson’s disease. Neurosci Lett. (1994) 172:151–4. doi: 10.1016/0304-3940(94)90684-X 8084523

[249] Mogi M Harada M Kondo T Riederer P Inagaki H Minami M. Interleukin-1 beta, interleukin-6, epidermal growth factor and transforming growth factor-alpha are elevated in the brain from parkinsonian patients. Neurosci Lett. (1994) 180:147–50. doi: 10.1016/0304-3940(94)90508-8 7700568

[250] Isik S Yeman Kiyak B Akbayir R Seyhali R Arpaci T. Microglia mediated neuroinflammation in parkinson’s disease. Cells. (2023) 12(7):1012. doi: 10.3390/cells12071012 37048085 PMC10093562

[251] Kurkowska-Jastrzebska I Balkowiec-Iskra E Ciesielska A Joniec I Cudna A Zaremba MM. Decreased inflammation and augmented expression of trophic factors correlate with MOG-induced neuroprotection of the injured nigrostriatal system in the murine MPTP model of Parkinson’s disease. Int Immunopharmacol. (2009) 9(6):781–91. doi: 10.1016/j.intimp.2009.03.003 19286483

[252] Banati RB Gehrmann J Schubert P Kreutzberg GW. Cytotoxicity of microglia. Glia. (1993) 7:111–8. doi: 10.1002/glia.440070117 8423058

[253] Sharifi H Mohajjel Nayebi A Farajnia S. 8-OH-DPAT (5-HT1A agonist) Attenuates 6-Hydroxy- dopamine-induced catalepsy and Modulates Inflammatory Cytokines in Rats. Iran J Basic Med Sci. (2013) 16(12):1270–5. PMC3933805 24570834

[254] Dominguez-Meijide A Villar-Cheda B Garrido-Gil P Sierrra-Paredes G Guerra MJ Labandeira-Garcia JL. Effect of chronic treatment with angiotensin type 1 receptor antagonists on striatal dopamine levels in normal rats and in a rat model of Parkinson’s disease treated with L-DOPA. Neuropharmacology. (2014) 76 Pt A:156–68. doi: 10.1016/j.neuropharm.2013.07.016 23973568

[255] McCoy MK Martinez TN Ruhn KA Szymkowski DE Smith CG Botterman BR. Blocking soluble tumor necrosis factor signaling with dominant-negative tumor necrosis factor inhibitor attenuates loss of dopaminergic neurons in models of Parkinson’s disease. J Neurosci. (2006) 26:9365–75. doi: 10.1523/JNEUROSCI.1504-06.2006 PMC3707118 16971520

[256] Wang YD Bao XQ Xu S Yu WW Cao SN Hu JP. A novel parkinson’s disease drug candidate with potent anti-neuroinflammatory effects through the src signaling pathway. J Med Chem. (2016) 59:9062–79. doi: 10.1021/acs.jmedchem.6b00976 27617803

[257] Samii A Etminan M Wiens MO Jafari S. NSAID use and the risk of Parkinson’s disease: systematic review and meta-analysis of observational studies. Drugs Aging. (2009) 26:769–79. doi: 10.2165/11316780-000000000-00000 19728750

[258] Gordon R Albornoz EA Christie DC Langley MR Kumar V Mantovani S. Inflammasome inhibition prevents α-synuclein pathology and dopaminergic neurodegeneration in mice. Sci Trans Med. (2018) 10:eaah4066. doi: 10.1126/scitranslmed.aah4066 PMC6483075 30381407

[259] Benner EJ Mosley RL Destache CJ Lewis TB Jackson-Lewis V Gorantla S. Therapeutic immunization protects dopaminergic neurons in a mouse model of Parkinson’s disease. Proc Natl Acad Sci. (2004) 101:9435–40. doi: 10.1073/pnas.0400569101 PMC438994 15197276

[260] Krashia P Cordella A Nobili A La Barbera L Federici M Leuti A. Blunting neuroinflammation with resolvin D1 prevents early pathology in a rat model of Parkinson’s disease. Nat Commun. (2019) 10:3945. doi: 10.1038/s41467-019-11928-w 31477726 PMC6718379

[261] Pajares M Rojo AI Manda G Boscá L Cuadrado A. Inflammation in parkinson’s disease: mechanisms and therapeutic implications. Cells. (2020) 9(7):1687. doi: 10.3390/cells9071687 32674367 PMC7408280

[262] Zella SM Metzdorf J Ciftci E Ostendorf F Muhlack S Gold R. Emerging immunotherapies for Parkinson disease. Neurol Ther. (2019) 8:29–44. doi: 10.1007/s40120-018-0122-z 30539376 PMC6534677

[263] Seo H-J Wang S-M Han C Lee S-J Patkar AA Masand PS. Curcumin as a putative antidepressant. Expert Rev Neurotherapeut. (2015) 15:269–80. doi: 10.1586/14737175.2015.1008457 25644944

[264] Arent SM Walker AJ Arent MA. “The effects of exercise on anxiety and depression”. In: Tenenbaum G Eklund RC, editors. Handbook of Sport Psychology (2020). doi: 10.1002/9781119568124.ch42

[265] Lurie DI. An integrative approach to neuroinflammation in psychiatric disorders and neuropathic pain. J Exp Neurosci. (2018) 12:1179069518793639. doi: 10.1177/1179069518793639 30127639 PMC6090491

[266] Ignácio ZM da Silva RS Plissari ME Quevedo J Réus GZ. Physical exercise and neuroinflammation in major depressive disorder. Mol Neurobiol. (2019) 56:8323–35. doi: 10.1007/s12035-019-01670-1 31228000

[267] Çınar E Tel BC Şahin G. Neuroinflammation in parkinson’s disease and its treatment opportunities. Balkan Med J. (2022) 39(5):318–33. doi: 10.4274/balkanmedj.galenos.2022.2022-7-100 PMC9469676 36036436

[268] Amirifar L Shamloo A Nasiri R de Barros NR Wang ZZ Unluturk BD. Brain-on-a-chip: Recent advances in design and techniques for microfluidic models of the brain in health and disease. Biomaterials. (2022) 285:121531. doi: 10.1016/j.biomaterials.2022.121531 35533441

[269] Drzezga A Barthel H Minoshima S Sabri O. Potential clinical applications of PET/MR imaging in neurodegenerative diseases. J Nuclear Med. (2014) 55:47S–55S. doi: 10.2967/jnumed.113.129254 24819417

